# Characterization of genetic structures of the *QepA3* gene in clinical isolates of *Enterobacteriaceae*

**DOI:** 10.3389/fmicb.2015.01147

**Published:** 2015-10-15

**Authors:** Dongguo Wang, Xitian Huang, Jiayu Chen, Yonghua Mou, Haijun Li, Liqin Yang

**Affiliations:** ^1^Department of Clinical Laboratory Medicine, Taizhou Municipal Hospital, Medical College of Taizhou University and the Institute of Molecular Diagnostics of Taizhou UniversityZhejiang, China; ^2^Department of Infectious Disease, Wenling Hospital, Wenzhou Medical UniversityZhejiang, China; ^3^Department of Laboratory Medicine, Medical College of Taizhou University and the Institute of Molecular Diagnostics of Taizhou UniversityZhejiang, China; ^4^Department of Hepatobiliary Surgery, Taizhou Municipal Hospital, Medical College of Taizhou UniversityZhejiang, China; ^5^Department of Neurology, Taizhou Municipal Hospital, Medical College of Taizhou UniversityZhejiang, China; ^6^Department of Quality Control, Taizhou Municipal Hospital, Medical College of Taizhou UniversityZhejiang, China

**Keywords:** plasmid-mediated quinolone resistance, *QepA3* gene, recombinant plasmid, genetic structure, gene variation

## Abstract

*QepA* is one of the genes that confer quinolone resistance in bacteria. The aim of this study was to analyze the genetic structures of plasmids that carry a *qepA3*, a recently discovered allele of *qepA* in *Enterobacteriaceae* clinical isolates. 656 non-redundant *Enterobacteriaceae* clinical isolates were screened for the *qepA3* gene and five isolates were identified to carry the gene. Plasmids were isolated from these isolates and were found to increase antibiotic resistance once the plasmids were transferred to *Escherichia coli*. These plasmids were subcloned and sequenced to analyze the genetic structures surrounding the *qepA3* gene. The results showed that the five plasmids had different genetic structures; two of the *qepA3*-containning isolates had either the *bla*_CTX-M-14_ or *bla*_TEM-12_ gene instead of the *bla*_TEM-1_ gene. The structures of both pKP3764 and pECL3786 have not been previously described. In comparison with pHPA, there were a number of changes in DNA sequences up- and down-stream of the *qepA3* gene. These findings provide better understanding of the genetic variations in *qepA3* and would be useful for diagnosis and control of quinolone resistance in clinical settings.

## Introduction

According to past work (Cattoir et al., 2008), three mechanisms have been described for plasmid- mediated quinolone resistance: *qnr* determinants (Mammeri et al., 2005; Nordmann and Poirel, 2005; Robicsek et al., 2006; Wang et al., 2011), aminoglycoside acetyltransferase *aac(6′)- Ib-cr* (Robicsek et al., 2006), and the *qepA* and *oqxAB* eﬄux pump genes, which confer decreased susceptibility to quinolones (Périchon et al., 2007; Yamane et al., 2007; Wong et al., 2015). The *qepA1* gene was first investigated in 2007 by two groups from Japan and Belgium (Périchon et al., 2007; Yamane et al., 2007). In 2008, the *qepA2* was discovered by a French research group (Cattoir et al., 2008). Currently, both the *qepA1* and *qepA2* genes have been reported worldwide (Liu et al., 2008; Park et al., 2009; Guillard et al., 2011; Ruiz et al., 2012; Chen et al., 2014). Recently, we identified a new *qepA* allele *qepA3* from in a Chinese patient (GenBank with accession number JQ064560). Although the human *qepA* has a fairly low prevalence in Korea (Kim et al., 2009; Park et al., 2009), it is commonly found in *Enterobacteriaceae* isolates from food- producing animals in China (Liu et al., 2008; Ma et al., 2009; Chen et al., 2014). Therefore, more surveillance is needed for *Enterobacteriaceae* harboring the *qnr*, *aac(6′)-Ib-cr* and *qepA*.

In this study, we investigated the *qepA* gene in 656 *Enterobacteriaceae* isolates from hospitalized patients and only 13 isolates were found positive. This result confirm that *qepA* has low prevalence (1.98%) (0.91% for *qepA1*, 0.31% for *qepA2* and 0.76% for *qepA3*) in patients of our area. However, five isolates were determined to harbor novel *qepA3* structures. These structure were characterized to provide better understanding of the gene for potential management of plasmid-mediated antibiotic resistance.

## Materials and Methods

### Bacterial Strains and Luria-Bertani (LB) Agar Plates

*Enterobacteriaceae* isolates EC3157, EC3587, CD4359, KP3764, and ECL3786 were identified using the Vitek 2 system (bioMérieux, France) according to the manufacturer’s instructions. *Escherichia co*li isolates EC3157 and EC3587 were isolated from the blood and sputum samples of ICU inpatients, respectively. CD4359, an isolate of *Citrobacter koseri*, was obtained from the sputum of an inpatient in Infection Unit, while KP3764, an isolate of *Klebsiella pneumoniae*, was from a blood sample of an ICU inpatient. ECL3786, an isolate of *Enterobacter cloacae*, was from the chest wound secretions of a cardiothoracic surgical inpatient. *E. coli JM109* was used as the host for cloning and an azide-resistant *E. coli* (strain J53) was used as the recipient strain for conjugation experiments. The LB1 agar medium for plasmid transformation contained 100 μL of isopropyl β-D-1-thiogalactopyranoside (IPTG, 24 mg/mL), 200 μL of 5-bromo-4-chloro- 3-indolyl β-D-galactopyranoside (X-Gal, 20 mg/mL) and 100 μL of ampicillin (Amp, 100 mg/mL) in 100 mL medium. LB2 agar plates were used for conjugation experiments and were supplemented with sodium azide (150 μg/mL) and ciprofloxacin (0.25 μg/mL).

### Susceptibility Testing

The minimum inhibitory concentrations (MICs) of eight antimicrobial agents (nalidixic acid, ofloxacin, ciprofloxacin, cefotaxime, ceftazidime, amikacin, and gentamicin) were determined using the MicroScan microdilution panel (Scott, USA) broth dilution method. *E. coli* ATCC 25922 and *Pseudomonas aeruginosa* ATCC 27853 were used as the controls. The results were interpreted according to the CLSI guidelines (Clinical and Laboratory Standards Institute, 2014).

### Plasmid Isolation and Sequence Analysis

Bacterial plasmid DNA was extracted using a plasmid extraction kit (TaKaRa, Japan) according to the manufacturer’s instructions. PCR amplifications were preformed using primers based on the pHPA (**Table 1**, Yamane et al., 2007). PCR was run for 3 min at 94°C followed by 30 cycles of 1 min of denaturing at 94°C and annealing at 56.9°C, with a final elongation of 10 min at 72°C on Life Veriti^®^ PCR machine (Invitrogen, USA). The total reaction volume was 20 μl containing 4 μL 5X PCR buffer, 0.4 μL of 10 mM dNTPs, 1 μL each of 10 μM primers and 0.2 μL Polymerase, with nuclease-free water filled up to 20 μL, and bacterial plasmids harboring the *qepA* gene as template. The amplicons were digested with *Dra*I and *Bam*HI (TaKaRa, Japan), ligated to linearized pMD19-T (TaKaRa, Japan), and transformed into *E. coli* JM109 competent cells. Plasmids DNA from Amp resistant colonies recovered on LB1 plate were sequenced.

**Table 1:** Primers used for PCR and sequencing.

Primer		Sequence (5′-3′)	Reference
tnpR	F	CGACACTGCCGATATGATCC	Park et al., 2009
	R	CGGGCAATACTGAGCTGATG	
TEM	F	ATAAAATTCTTGAAGACGAAA	Lee et al., 2003
	R	GACAGTTACCAATGCTTAATC	
rmtB	F	CCCAAACAGACCGTAGAGGC	Lee et al., 2006
	R	CTCAAACTCGGCGGGCAAGC	
qepA	F	AGCAGCGCGCTGAATCCA	This study
	R	CGAACCCAGTGGACATAA	
qepA (sequencing)	F	AGC AGCGCGCTGAATCCA	This study
	R	CTTCCTGCCCGAGTA TCG TG	
intI1	F	GCCTTGCTGTTCTTCTACGG	Han et al., 2004
	R	GATGCCTGCTTGTTCTACGG	
tnpA	F	GGCGGGATCTGCTTGTAGAG	Han et al., 2004
	R	CTCCGGAGATGTCTGGCTTACT	
dfr2	F	TTGGGCTTCACCAGAGTATCAAGTT	Cattoir et al., 2008
	R	GCTGTGGACGGTGCCGCATGATTTG	
CTX-M-14	F	GAAAGAGAGTGCAACGGATG	This study
	R	ATTGGAAAGGGTTCATCACC	

### Conjugation

The five isolates were conjugated with *E. coli* J53 as described previously (Wang et al., 2003). In the conjugation experiments, the isolates were used as donors and azide-resistant *E. coli* J53 as the recipient strain by filter mating. Transconjugants were selected on the LB2 agar plates. MICs for the donors, transconjugants, and recipients were also measured as described above.

## Results

### Plasmid Isolation and Characterization

**FIGURE 1:**
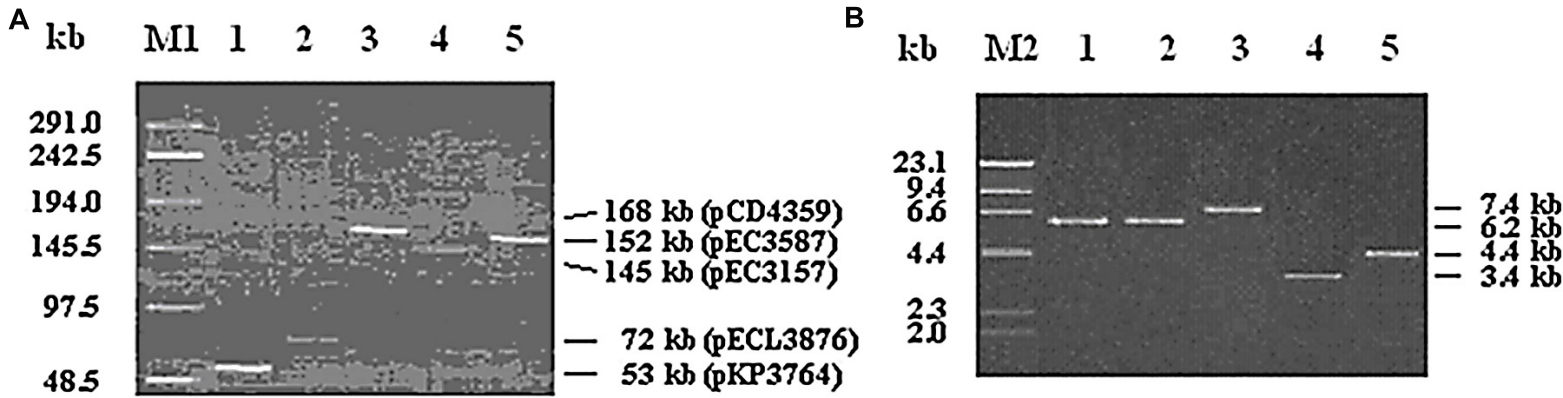
**Electrophoresis of plasmids isolated from clinical isolates and the recombinant plasmids used for sequence analysis.** The left columns indicate the DNA markers while the right column denote the plasmid sizes. **(A)** Lane M1, a λ DNA marker (Amersham Biosciences, USA); Lanes 1–5, plasmids pKP3764, pECL3876, pEC3157, pEC3587, and pCD4359. **(B)** Lane M2, λ *Hind*III DNA marker (Amersham Biosciences, USA); Lanes 1–5, recombinant plasmids pECD2, pECD4, pCDD2, pKPD1, and pECLD1.

Plasmids were isolated from the five *Enterobacteriaceae* isolates and separated on 0.7% agarose gel by gel electrophoresis. Results showed that plasmids pKP3764, pECL3876, pEC3157, pEC3587, and pCD4359 from isolates KP3764, pECL3876, EC3157, EC3587, and CD4359 were estimated to be about 53 kb, 72 kb, 145, 152, and 168 kb, respectively (**Figure 1A**).

**FIGURE 2:**
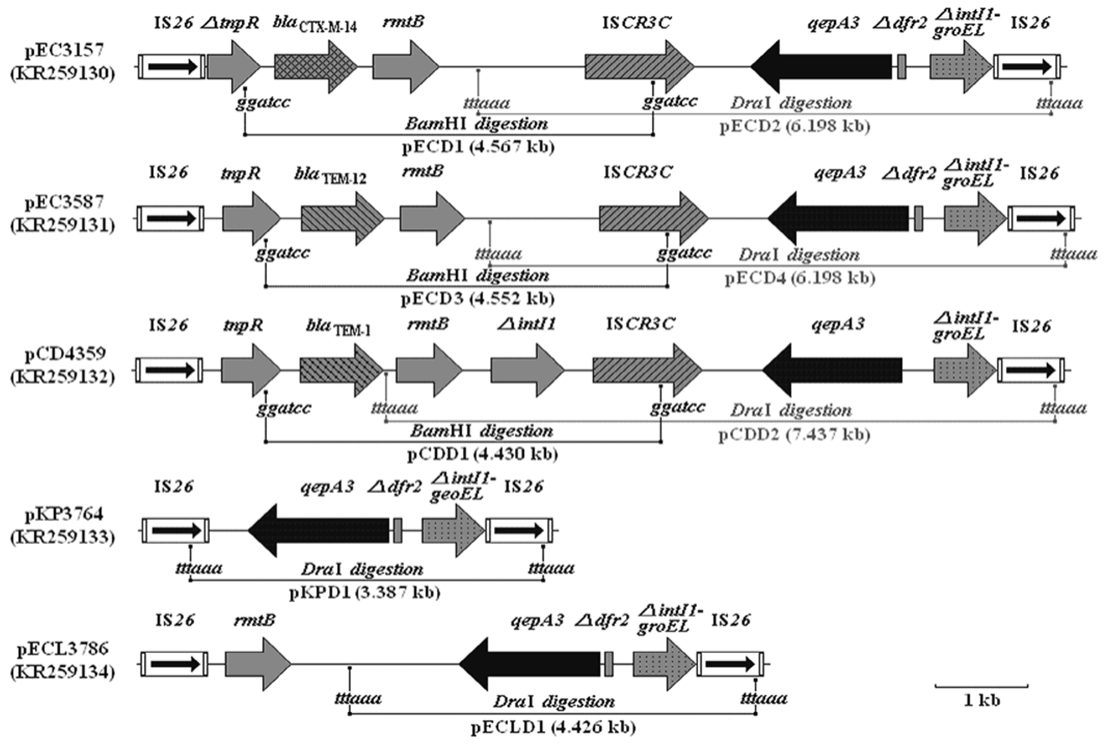
**Genetic structures surrounding the *qepA3* gene in pEC3157, pEC3587, pCD4359, pKP3764, and pECL3786.** The numbers in parentheses are GenBank accession numbers; “*ggatcc*” and “*tttaaa*” indicate restriction sites; Δ *(delta)* denotes truncated gene; Δ*intI1-groEL* denotes the truncated *intI1* and *groEL* fusion gene. The figures in the parentheses following the names of the plasmids denote the plasmid size.

The *qep A*-containing sequences in the five strains were amplified, digested with *Dra*I and/or *Bam*HI and subcloned in pMD19-T to generate recombinant plasmids pECD1, pECD2, pECD3, pECD4, pCDD2, pKPD1, and pECLD1 (**Figure 1B**). The inserts in these plasmids were sequenced and analyzed for *qep A* and its flanking structures (GenBank accession numbers KR259130, KR259131, KR259132, KR259133, and KR259134, Supplementary Material) (**Figure 2**). The results showed that in addition to *qepA3*, which was present in all plasmids, the inserts from pEC3157 and pCD4359 contained the *bla*_CTX-M-14_ and *rmtB* genes; the inserts from pEC3587 had the *bla*_TEM-12_ gene; inserts from all plasmids except pCD4359 had the truncated *dfr2* gene; the inserts from pCD4539 contained the *bla*_TEM-1_ and *rmtB* genes; the insert from pKPD1 and pECLD1 contained truncated *dfr2* gene. No other resistant genes were found in these sequences.

### Antibiotic Susceptibility

Through conjugation, the plasmids from the five isolates (donors) were transferred to *E. coli* J53 (recipients). The MIC values for cefotaxime and ceftazidime in the transconjugants with the isolates EC3157, EC3587, and CD4359 were much higher than those of the recipient *E. coli* J53 (**Table 2**). Similarly, the MIC values for nalidixic acid, norfloxacin, ofloxacin, and ciprofloxacin in the transconjugants with the five isolates were generally higher than those of *E. coli* J53 (**Table 2**). Contrastingly, the MIC values nalidixic acid, ofloxacin, ciprofloxacin, amikacin, and gentamicin in the transconjugant involving the EC3157, EC3587, CD4357, and ECL3786 were generally higher than those of *E. coli* J53 (recipient) (**Table 2**). These results demonstrated that the antibiotic resistant genes are located in the plasmids.

**Table 2:** Minimum inhibitory concentrations of antimicrobial agents for donors, transconjugants, and their recipients.

Stain	MIC (μg/mL)							
	NAL	NOR	OFL	CIP	CTX	CAZ	AMK	GM
**Donor**								
EC3157	128	8	4	2	128	4	64	128
EC3587	128	16	4	4	32	4	128	>128
CD4359	128	16	4	4	32	2	64	128
KP3764	128	8	4	2	0.25	0.25	0.50	0.125
ECL3786	32	8	2	2	0.125	0.125	64	128
**Transconjugant**								
EC3157*- Escherichia coli* J53	8	2	1	0.50	8	1	16	32
EC3587*- E. coli* J53	16	4	1	1	4	1	32	64
CD4359*- E. coli* J53	16	4	1	1	4	1	16	64
KP3764*- E. coli* J53	16	2	1	0.50	0.125	0.125	0.50	0.063
ECL3786*- E. coli* J53	8	2	0.50	0.50	0.063	0.125	16	32
**Recipient**								
*E. coli* J53	4	0.25	0.032	0.016	0.063	0.25	0.50	0.063

*NAL, nalidixicacid; OFL, ofloxacin; CIP, ciprofloxacin; CTX, cefotaxime; CAZ, ceftazidime; AMK, amikacin; GEN, gentamicin; NOR, norfloxacin.*

## Discussion

Previous studies have shown that quinolone and fluoroquinolone resistance genes in *E. coli* and *Klebsiella* isolates are located on plasmids, which often carry other antimicrobial resistant genes, and can be transferred to other strains by conjugation or transformation (Ruiz et al., 2012). Since the discovery of *qepA1* and *qepA2* in 2007 and 2008, the genetic environment and location of the *qepA* genes in the plasmid pHPA have both been well-established (Périchon et al., 2007; Yamane et al., 2007; Cattoir et al., 2008). In this study, five *Enterobacteriaceae* isolates carrying the *qepA3* and surrounding genes were investigated, and are found to have different genetic structures (**Figure 2**) surrounding *qepA3.*

The plasmid pCD4359 from isolate CD4359 showed a similar structure to pHPA, except for the *qepA* allele and a truncated *dfr2*, a gene closely related to vertebrate FGF-receptor. The plasmids pEC3587 and pEC3157 were different from pHPA, where *bla*_TEM-1_ in pHPA was replaced by *bla*_TEM-12_ or *bla*_CTX-M-14_, respectively (**Figure 2**). *bla*_CTX-M-14_,*bla*_TEM-1,_ and *bla*_TEM-12_ code β-lactamase and are important determinants of drug resistance. In addition, in pCD4359, there is an additional *ΔintI1* downstream of the *rmtB which* confers high-level resistance to all aminoglycoside and missing *Δdfr2*. The sequence between IS*26* and *tnpR* was also truncated. Furthermore, *rmtB* or *ISCR3C* are not linked with *qepA3* directly in pKP3764 as reported previously (Périchon et al., 2007; Yamane et al., 2007; Cattoir et al., 2008; Kim et al., 2009; Rocha-Gracia et al., 2010; Cao et al., 2014).

In pEC3587, the *bla*_TEM-12_ gene took the place of *bla*_TEM-1_ and had a truncated *dfr2* gene as compared with pHPA. These results are also different from previous reports (Périchon et al., 2007; Yamane et al., 2007; Cattoir et al., 2008; Kim et al., 2009; Rocha-Gracia et al., 2010; Cao et al., 2014). Surprisingly, in pKP3764 there are three genes *qepA3*, truncated *dfr2*, and truncated *intI1-groEL* fusion gene between the two IS*26* insertions, while in pKP3764 and pECL3786, therefore are four genes: *rmtB*, *qepA3*, truncated *dfr2*, and truncated *intI1-groEL* fusion genes. To date, these genetic structures have not been identified in previous studies (Kim et al., 2009; Rocha-Gracia et al., 2010; Cao et al., 2014). Moreover, pKP3764 is the only plasmid that does not contain the *rmtB* gene among the five plasmids studied in this study. According to a previous report, 58.3% (28/48) of *rmtB*-positive *E. coli* isolates harbored the *qepA* gene (Grape et al., 2005). Whether or not these results suggest a strong link between *qepA* and *rmtB* remains to be investigated. In these five genetic structures (**Figure 2**), the *qepA3* gene and its downstream genes were nearly all identical except for the truncated *dfr2* deletion in pCD4359. After pEC3157 and pEC3587 were digested by the *Dra*I, the size of both products were identical at approximate 6.2 kb. It should be noted that it is also possible that the IS*CR3C* is not a stable link between IS*CR3C* and the *qepA* gene.

*QepA* gene is a quinolone pump gene that confers resistance to nalidixic acid and norfloxacin. Stains carrying the gene may be resistant or sensitive to ofloxacin and ciprofloxacin with increased MIC. In the conjugation experiments, we found that *E. coli* cells with pEC3587 and pCD4359 were resistant to penicillins and aminoglycoside antibiotics drugs such as amikacin, gentamicin, gentamicin, and tobramycin. They were tolerant or sensitive to ofloxacin and ciprofloxacin, but the MICs were increased. These results indicate that there might be synergistic effect against antibiotics when the *gepA*, *blaCTX-M 14*, and *rmtB* are present in the same plasmids, leading to multidrug resistance.

Taken together, our works suggest that there are sequence variations surrounding the *qepA3* even if a limited number of isolates are analyzed, and it is likely that more variations would exist that may impact the resistance profiles, and subsequently the clinical implications of the bacteria. More studies are needed to link these structure variation to resistance profiles and potential clinical outcomes.

## Conclusion

The novel genetic structures surrounding the *qepA3* gene have been discovered in the isolates obtained from five patients in China although the prevalence for the *qepA* allele in hospital patients are low. One of the isolates is linked to the non-*rmtB*- or non- IS*CR3C*- producing genetic structure in the *qepA3* genetic environment. Moreover, the *bla*_CTX-M-14_ or *bla*_TEM-12_ genes are found to be associated with the *qepA3* gene in these structures instead of the *bla*_TEM-1_ gene. These results provide new insight into the variation in genetic environments of the *qepA3* gene and would be useful for further investigation of the clinical implications in antibiotic resistance management.

## Conflict of Interest Statement

The authors declare that the research was conducted in the absence of any commercial or financial relationships that could be construed as a potential conflict of interest.

## References

[B1] Cao X. Xu X. Zhang Z. Shen H. Chen J. Zhang K. (2014). Molecular characterization of clinical multidrug-resistant *Klebsiella pneumoniae* isolates. *Ann. Clin. Microbiol. Antimicrob.* 13:16 10.1186/1476-0711-13-16 PMC4030571 24884610

[B2] Cattoir V. Poirel L. Nordmann P. (2008). Plasmid-mediated quinolone resistance pump qepA2 in an *Escherichia coli* isolate from France. *J. Antimicrob. Chemother.* 52 3801–3804. 10.1016/j.diagmicrobio.2009.07.006 PMC2565908 18644958

[B3] Chen X. He L. Li Y. Zeng Z. Deng Y. Liu Y. (2014). Complete sequence of a F2: A-: B- plasmid pHN3A11 carrying rmtB and qepA, and its dissemination in China. *Vet. Microbiol.* 174 267–271. 10.1016/j.vetmic.2014.08.023 25236985

[B4] Clinical and Laboratory Standards Institute (2014). *Performance Standards for Antimicrobial Susceptibility Testing; Twenty-Four Informational Supplement. M100–S24.* Wayne, PA: Clinical and Laboratory Standards Institute.

[B5] Grape M. Farra A. Kronvall G. Sundstrëm L. (2005). Integrons and gene cassettes in clinical isolates of co-trimoxazole-resistant gram-negative bacteria. *Clin. Microbiol. Infect.* 11 185–192. 10.1111/j.1469-0691.2004.01059.x 15715715

[B6] Guillard T. Moret H. Brasme L. Carlier A. Vernet-Garnier V. Cambau E. (2011). Rapid detection of qnr and qepA plasmid-mediated quinolone resistance genes using real-time PCR. *Diagn. Microbiol. Infect. Dis.* 70 253–259. 10.1016/j.diagmicrobio.2011.01.004 21596225

[B7] Han H. S. Koh Y. J. Hur J.-S. Jung J. S. (2004). Occurrence of the strA-strB Streptomycin resistance genes in *Pseudomonas* species isolated from Kiwifruit plants. *J. Microbiol.* 42 365–368. 15650697

[B8] Kim H. B. Park C. H. Kim C. J. Kim E. C. Jacoby G. A. Hooper D. C. (2009). Prevalence of plasmid-mediated quinolone resistance determinants over a 9-year period. *Antimicrob. Agents Chemother.* 53 639–645. 10.1128/AAC.01051-08 19064896 PMC2630623

[B9] Lee H. Yong D. Yum J. H. Roh K. H. Lee K. Yamane K. (2006). Dissemination of 16 S rRNA methylase-mediated highly amikacin-resistant isolates of *Klebsiella pneumoniae* and *Acinetobacter baumannii* in Korea. *Diagn. Microbiol. Infect. Dis.* 56 305–312. 10.1016/j.diagmicrobio.2006.05.002 16822637

[B10] Lee K. Yong D. Yum J. H. Kim H. H. Chong Y. (2003). Diversity of TEM-52 extended-spectrum β-lactamase-producing non-typhoidal *Salmonella* isolates in Korea. *J. Antimicrob. Chemother.* 52 493–496. 10.1093/jac/dkg385 12917235

[B11] Liu J. H. Deng Y. T. Zeng Z. H. Gao J. H. Chen L. Arakawa Y. (2008). Coprevalence of plasmid-mediated quinolone resistance determinants QepA, Qnr, and AAC(6’)- Ib-cr among 16 S rRNA methylase RmtB-producing *Escherichia coli* isolates from pigs. *Antimicrob. Agents Chemother.* 52 2992–2993. 10.1128/AAC.01686-07 18490500 PMC2493129

[B12] Ma J. Zeng Z. Chen Z. Xu X. Wang X. Deng Y. (2009). High prevalence of plasmid-mediated quinolone resistance determinants qnr, aac(6’)-Ib-cr, and qepA among ceftiofur-resistant Enterobacteriaceae isolates from companion and food-producing animals. *Antimicrob. Agents Chemother.* 53 519–524. 10.1128/AAC.00886-08 18936192 PMC2630616

[B13] Mammeri H. Van De Loo M. Poirel L. Martinez-Martinez L. Nordmann P. (2005). Emergence of plasmid-mediated quinolone resistance in *Escherichia coli* in Europe. *Antimicrob. Agents Chemother.* 49 71–76. 10.1128/AAC.49.1.71-76.2005 15616277 PMC538905

[B14] Nordmann P. Poirel L. (2005). Emergence of plasmid-mediated resistance to quinolones in Enterobacteriaceae. *J. Antimicrob. Chemother.* 56 463–469. 10.1093/jac/dki245 16020539

[B15] Park Y.-J. Jin K. Y. Kim S.-I. Lee K. Arakawa Y. (2009). Accumulation of plasmid-mediated fluoroquinolone resistance genes, qepA and qnrS1, in Enterobacter aerogenes co-producing RmtB and class A β-lactamase LAP-1. *Ann. Clin. Lab. Sci.* 39 55–59. 19201742

[B16] Périchon B. Courvalin P. Galimand M. (2007). Transferable resistance to aminoglycosides by methylation of G1405 in 16S rRNA and to hydrophilic fluoroquinolones by qepA-mediated eﬄux in *Escherichia coli*. *Antimicrob. Agents Chemother.* 51 2464–2469. 10.1128/AAC.00143-07 17470656 PMC1913276

[B17] Robicsek A. Jacoby G. A. Hooper D. C. (2006). The worldwide emergence of plasmid-mediated quinolone resistance. *Lancet Infect. Dis.* 6 629–640. 10.1016/S1473-3099(06)70599-0 17008172

[B18] Rocha-Gracia R. Ruiz E. Romero-Romero S. Lozano-Zarain P. Somalo S. Palacios-Hernández J. (2010). Detection of the plasmid- borne quinolone resistance determinant qepA1 in a CTX-M-15-producing *Escherichia coli* strain from Mexico. *J. Antimicrob. Chemother.* 65 169–171. 10.1093/jac/dkp418 19910326

[B19] Ruiz E. Sáenz Y. Zarazaga M. Rocha-Gracia R. Martínez-Martínez L. Arlet G. (2012). Qnr, aac(6’)-Ib-cr and qepA genes in *Escherichia coli* and *Klebsiella* spp: genetic environments and plasmid and chromosomal location. *J. Antimicrob. Chemother.* 67 886–897. 10.1093/jac/dkr548 22223228

[B20] Wang D. Wang H. Qi Y. Liang Y. Zhang J. Yu L. (2011). Novel variants of the qnrB gene, qnrB31 and qnrB32, in *Klebsiella pneumoniae*. *J. Med. Microbiol.* 60 1849–1852. 10.1099/jmm.0.034272-0 21816942

[B21] Wang M. Tran J. H. Jacoby G. A. Zhang Y. Wang F. Hooper D. C. (2003). Plasmid-mediated quinolone resistance in clinical isolates of *Escherichia coli* from Shanghai, China. *Antimicrob. Agents Chemother.* 47 2242–2248. 10.1128/AAC.47.7.2242-2248.2003 12821475 PMC161834

[B22] Wong M. H. Chan E. W. Chen S. (2015). Evolution and dissemination of oqxAB- like eﬄux pumps, an emerging quinolone resistance determinant among members of Enterobacteriaceae. *Antimicrob. Agents Chemother.* 59 3290–3297. 10.1128/AAC.00310-15 25801572 PMC4432112

[B23] Yamane K. Wachino J. Suzuki S. Kimura K. Shibata N. Kato H. (2007). New plasmid-mediated fluoroquinolone eﬄux pump, qepA, found in an *Escherichia coli* clinical isolate. *Antimicrob. Agents Chemother.* 51 3354–3360. 10.1128/AAC.00339-07 17548499 PMC2043241

