# Supplementary material for: Characterization of genetic structures of the QepA3 gene in clinical isolates of Enterobacteriaceae
Source: Front Microbiol. 2015 Oct 15;6:1147. doi: 10.3389/fmicb.2015.01147 (PMC4606065; doi:10.3389/fmicb.2015.01147)
Supplement: Supplementary file 1 [file Presentation_1.PDF]

The following data will be released periodically to public on 13-SEP-2015.

GenBank flat files:

```
LOCUS       KR259130               10236 bp    DNA     circular BCT 08-JUL-2015
DEFINITION  Escherichia coli strain EC3157 plasmid pEC3157, complete sequence.
ACCESSION   KR259130
VERSION     KR259130
KEYWORDS    .
SOURCE      Escherichia coli
  ORGANISM  Escherichia coli
            Bacteria; Proteobacteria; Gammaproteobacteria; Enterobacteriales;
            Enterobacteriaceae; Escherichia.
REFERENCE   1  (bases 1 to 10236)
  AUTHORS   Wang,D., Huang,X. and Chen,J.
  TITLE     A plasmid from the isolate EC3157 of Escherchia coli containing
            relevant genes haboring 16S rRNA methylases, blaCTX-M-14 and novel
            qepA3 genes from patient's blood in Taizhou municipal hospital of
            China
  JOURNAL   Unpublished
REFERENCE   2  (bases 1 to 10236)
  AUTHORS   Wang,D., Huang,X. and Chen,J.
  TITLE     Direct Submission
  JOURNAL   Submitted (22-APR-2015) Department of Clinical Lab Medicine,
            Taizhou Municipal Hospital and Institute of Molecular Diagnostics
            of Taizhou University, 381 East Rd. of Zhongshan in Jiaojiang of
            Taizhou, Taizhou, Zhejiang 318000, China
COMMENT     ##Assembly-Data-START##
            Sequencing Technology :: Sanger dideoxy sequencing
            ##Assembly-Data-END##
FEATURES             Location/Qualifiers
     source          1..10236
                     /organism="Escherichia coli"
                     /mol_type="genomic DNA"
                     /strain="EC3157"
                     /host="Homo sapiens"
                     /db_xref="taxon:562"
                     /plasmid="pEC3157"
                     /country="China: Taizhou municipal hospital of Zhejiang
                     province"
                     /lat_lon="28 N 122 E"
                     /collection_date="09-Apr-2013"
                     /collected_by="Dongguo Wang"
                     /note="genotype:
                     tnpA;tnpR;blaCTX-M-14;rmtB;tnpA;qepA3;dfr2;intI1-
                     groEL;tnpA"
     gene            42..758
                     /gene="tnpA"
                     /note="insertion sequence:IS26; tnpA gene; transposase"
                     /pseudo
     gene            811..1368
                     /gene="tnpR"
                     /note="truncated resolvase; tnpR gene; TnpR"
                     /pseudo
     gene            1551..2426
                     /gene="blaCTX-M-14"
     CDS             1551..2426
                     /gene="blaCTX-M-14"
                     /note="beta-lactamase"
                     /codon_start=1
```

```
                     /transl_table=11
                     /product="CTX-M-14"
                     /protein_id="AKP49175"
                     /translation="MVTKRVQRMMFAAAACIPLLLGSAPLYAQTSAVQQKLAALEKSS
                     GGRLGVALIDTADNTQVLYRGDERFPMCSTSKVMAAAAVLKQSETQKQLLNQPVEIKP
                     ADLVNYNPIAEKHVNGTMTLAELSAAALQYSDNTAMNKLIAQLGGPGGVTAFARAIGD
                     ETFRLDRTEPTLNTAIPGDPRDTTTPRAMAQTLRQLTLGHALGETQRAQLVTWLKGNT
                     TGAASIRAGLPTSWTVGDKTGSGDYGTTNDIAVIWPQGRAPLVLVTYFTQPQQNAESR
                     RDVLASAARIIAEGL"
gene                 2596..3351
                     /gene="rmtB"
CDS                  2596..3351
                     /gene="rmtB"
                     /codon_start=1
                     /transl_table=11
                     /product="16S rRNA methylase"
                     /protein_id="AKP49176"
                     /translation="MNINDALTSILASKKYRALCPDTVRRILTEEWGRHKSPKQTVEA
                     ARTRLHGICGAYVTPESLKAAAAALSAGDVKKALSLHASTKERLAELDTLYDFIFSAE
                     TPRRVLDIACGLNPLALYERGIASVWGCDIHQGLGDVITPFAREKDWDFTFALQDVLC
                     APPAEAGDLALIFKLLPLLEREQAGSAMALLQSLNTPRMAVSFPTRSLGGRGKGMEAN
                     YAAWFEGGLPAEFEIEDKKTIGTELIYLIKKNG"
gene                 4950..6152
                     /gene="tnpA"
                     /note="insertion sequence:ISCR3; tnpA gene; transposase
                     ISCR3C"
                     /pseudo
gene                 complement(6805..8340)
                     /gene="qepA3"
CDS                  complement(6805..8340)
                     /gene="qepA3"
                     /note="quinolone efflux pump"
                     /codon_start=1
                     /transl_table=11
                     /product="QepA3"
                     /protein_id="AKP49177"
                     /translation="MSATLHDTAADRRKATRREWIGLAVVALPCLVYAMDLTVLNLAL
                     PVLSRELQPSSAQLLWILDIYGFFVAGFLITMGTLGDRIGRRRLLLIGAAFFAFASVL
                     AALADTAALLIAARALLGLAGATIAPSTMALVRNMFHDPRQRQFAIGVWIAAFSLGSA
                     IGPLVGGVLLEFFHWGAVFWLNVPVMLLTLALGPRFLPEYRDPDAGHLDLASVLLSLA
                     AVLLTIYGLKQLAEHGEGLASMAALLAGLAVGALFLRRQGHIAYPLLDLRLFAHALFR
                     AALAAYALAALAMFGVYIFMTQYLQLVLGLSPLQAGLATLPCSLCFVIGSLLSPQLAA
                     RWPAARILVVGLSAAAFGFAVLGLGQGLWWLVPATIVKGLGLAPVFTIGNEIIITSAP
                     SERAGAASALSETVSEFSGALGIALFGSVGLVVYRQALTSAALPGLPADALQTAGASL
                     GGAVHLADTLPAWQGAALLAAARAGFTDALQATAWAGAVLVLVAAGLVARLLRKRPAL
                     ASG"
gene                 8399..8509
                     /gene="dfr2"
CDS                  8399..8509
                     /gene="dfr2"
                     /note="pseudo; trimethoprim dihydrofolate reductase"
                     /codon_start=1
                     /transl_table=11
                     /product="Dfr2"
                     /protein_id="AKP49178"
                     /translation="MPSMATPIRIQARVTRMPVARRTSGGTVVAGQQLKY"
gene                 8735..9433
                     /gene="fusion groEL/intI1"
                     /note="similar to chaperonin/integrase fusion protein;
                     fusion gene groEL/intI1; integrase fusion protein
                     groEL/intI1"
```

```
                     /pseudo
     gene            9468..10184
                     /gene="tnpA"
                     /note="tnpA gene; transposase"
                     /pseudo
ORIGIN
        1 caaatagtcg gtggtgataa acttatcatc cccttttgct gatggagctg cacatgaacc
       61 cattcaaagg ccggcatttt cagcgtgaca tcattctgtg ggccgtacgc tggtactgca
      121 aatacggcat cagttaccgt gagctgcagg agatgctggc tgaacgcgga gtgaatgtcg
      181 atcactccac gatttaccgc tgggttcagc gttatgcgcc tgaaatggaa aaacggctgc
      241 gctggtactg gcgtaaccct tccgatcttt gcccgtggca catggatgaa acctacgtga
      301 aggtcaatgg ccgctgggcg tatctgtacc gggccgtcga cagccggggc cgcactgtcg
      361 atttttatct ctcctcccgt cgtaacagca aagctgcata ccggtttctg ggtaaaatcc
      421 tcaacaacgt gaagaagtgg cagatcccgc gattcatcaa cacggataaa gcgcccgcct
      481 atggtcgcgc gcttgctctg ctcaaacgcg aaggccggtg cccgtctgac gttgaacacc
      541 gacagattaa gtaccggaac aacgtgattg aatgcgatca tggcaaactg aaacggataa
      601 tcggcgccac gctgggattt aaatccatga agacggctta cgccaccatc aaaggtattg
      661 aggtgatgcg tgcactacgc aaaggccagg cctcagcatt ttattatggt gatcccctgg
      721 gcgaaatgcg cctggtaagc agagtttttg aaatgtaagg cctttgaata agacaaaagg
      781 ctgcctcatc gctaactttg caacagtgcc atgcgacttt ttggttacgc tcgggtctca
      841 accagtcagc agtctcttga tcttcaggtc agagcactca aagacgcagg tgtgaaagca
      901 aaccgtatat ttaccgataa ggcatccggc agttcaacag accgggaagg gctggatttg
      961 ctgaggatga aggtggagga aggtgatgtc attctggtta agaagctcga ccgtcttggc
     1021 cgcgacactg ccgatatgat ccaactgata aaggaatttg acgctcaggg cgtggcagtc
     1081 cggttcattg atgacgggat cagtaccgac ggtgatatgg ggcaaatggt ggtcaccatc
     1141 ctgtcggctg tggcacaggc tgaacgccgg aggatcctag aacgcacgaa tgagggccga
     1201 caggaagcaa agctgaaagg aatcaaattt ggccgcaggc gtaccgtgga caggaacgtc
     1261 gtgctgacgc ttcatcagaa gggcactggt gcaacggaaa ttgctcatca gctcagtatt
     1321 gcccgctcca cggtttataa aattcttgaa gacgaaaggg cctcgtgata cgcctatttt
     1381 tataggttaa tgtcatgata ataatggttt cttagacgtc aggtggcact tttcggggaa
     1441 atgtgcgcgg aacccctatt tgtttatttt tctaaataca ttcaaatatg tatccgctca
     1501 tgagacaata accctggtaa atgcttcaat aatattgaaa aaggaagagt atggtgacaa
     1561 agagagtgca acggatgatg ttcgcggcgg cggcgtgcat tccgctgctg ctgggcagcg
     1621 cgccgcttta tgcgcagacg agtgcggtgc agcaaaagct ggcggcgctg gagaaaagca
     1681 gcggagggcg gctgggcgtc gcgctcatcg ataccgcaga taatacgcag gtgctttatc
     1741 gcggtgatga acgctttcca atgtgcagta ccagtaaagt tatggcggcc gcggcggtgc
     1801 ttaagcagag tgaaacgcaa aagcagctgc ttaatcagcc tgtcgagatc aagcctgccg
     1861 atctggttaa ctacaatccg attgccgaaa aacacgtcaa cggcacaatg acgctggcag
     1921 aactgagcgc ggccgcgttg cagtacagcg acaataccgc catgaacaaa ttgattgccc
     1981 agctcggtgg cccgggaggc gtgacggctt ttgcccgcgc gatcggcgat gagacgtttc
     2041 gtctggatcg cactgaacct acgctgaata ccgccattcc cggcgacccg agagacacca
     2101 ccacgccgcg ggcgatggcg cagacgttgc gtcagcttac gctgggtcat gcgctgggcg
     2161 aaacccagcg ggcgcagttg gtgacgtggc tcaaaggcaa tacgaccggc gcagccagca
     2221 ttcgggccgg cttaccgacg tcgtggactg tgggtgataa gaccggcagc ggcgactacg
     2281 gcaccaccaa tgatattgcg gtgatctggc cgcagggtcg tgcgccgctg gttctggtga
     2341 cctattttac ccagccgcaa cagaacgcag agagccgccg cgatgtgctg gcttcagcgg
     2401 cgagaatcat cgccgaaggg ctgtaactgt cagaccaagt ttactcatat atactttaga
     2461 ttgatttaaa acttcatttt taatttaaaa ggatctaggt gaagatcctt tttgataatc
     2521 tcatgaccaa aatcccttaa cgtgagtttt cgttccactg agcgtcagac cccgaaaacg
     2581 atttaggaga caccgatgaa catcaacgat gccctcacct ccatcctggc ctcaaaaaaa
     2641 taccgcgccc tttgcccgga taccgtgcgg cgcatcctga ctgaggaatg ggggcggcat
     2701 aaatcccccca aacagaccgt agaggctgca cgcacccggc tgcatggaat ttgcggggca
     2761 tatgtcaccc cggaatcgct caaggctgct gccgccgcgc tttctgcggg cgatgtaaaa
     2821 aaggcattgt cgctgcatgc ctccaccaag gagcgactgg ccgagctgga taccctgtac
     2881 gattttatct tttcagccga aactccccgc cgcgtgctgg atatcgcctg cggtcttaac
     2941 cccttggcgc tatacgagcg cggcattgca tccgtgtggg gctgtgatat ccaccaggga
     3001 ttgggggatg tcatcacccc ctttgctagg gaaaaagatt gggattttac ctttgccctg
     3061 caggatgtgc tgtgtgcgcc gcccgccgaa gccggcgacc tggcgctgat ttttaagctt
     3121 ttgcccctgc tggagcggga gcaggccggt tctgccatgg cacttttaca atccctcaat
     3181 accccgcgca tggctgtcag ctttcccacg cgtagtttag gcgggcgtgg aaaaggcatg
     3241 gaggcgaact acgccgcatg gttcgagggc ggcttgcccg ccgagtttga gattgaggat
```

3301 aaaaagacca tcggaacaga acttatatac ttgataaaaa agaatggata agccaatcag
3361 aaggcaaatc atctaaaaaa gaggaacgaa aaatattttt cgttcctctt ttgttctatc
3421 gaacaagcac actacgactg ctgtttttga agcagccttt tatagctcag ctcgatgccc
3481 aacgccccca gcggcgctgt aagtataatg gcaagaaccg ccaccgcaag tatggtttcc
3541 ccactgccaa gccccatcgc cagcggaata gcgccgattg cagcctgcac cgttgccttg
3601 ggcagatagg caatcatgca gaacaggcgc tccttccggg ataggtctgt acccagcgta
3661 ctcatccaca cgcccagcat gcgaaacagc agcgcagctg tgatcagcag catgccgctt
3721 agcccggcgg agaaaaggta acggatgttg accgttgcac ccaccagcac aaacagccag
3781 atttctgccg ccacccaaag ctttgaaaat ttgccggata tacgctttgc aaccggggcg
3841 ttggttttta aaagggtgac gcccatcccc atcaccgcca gcaatccgga aaatagcgcc
3901 ctttcgccca gggttttctc caacgcaacc agcgcaaagg atgcgcccag cagcaggagc
3961 accttgatcg tatcacgcat atgtacccgc ttaaaaaggg ctgccggccg gcaaggtgct
4021 gtgcacggat ctgccctggc agggcatcaa cgaagaccag aacctgggca tcgccatcac
4081 ccgccgtgcg ctggaagccc cgctgcgcgc catcgtggcc aacgccggtg aagaaccgag
4141 cgtgatcgtg gccaacgtca aggccggcga aggcagctac ggctacaacg ccgccaccgg
4201 cgagttcggc gacatgatcg ccatgggcat cctggacccg accaaggtga cccgctcggc
4261 cctgcagcac gccgcttccg tcgccggcct tgcgatcacg accgaagtgg tcgtggccga
4321 agtgccgaag aaggaagagc cggccatgcc gggtgctggc ggtatgggcg gcatgggcgg
4381 catggatttc tgatccggtt ggcccggtcg tcagggaacc ggaccgcgcc agcgcggtcc
4441 gatcccggca acgacccgac atcaaggccc caaggacggg gccggagccc ggcagcgatg
4501 ccgggctttt tgttgtgccc gcgccgcggc aatgtctgac gcgaagatca gaacgcaccg
4561 atacgaacgt gcgaacacag gcgcaacact gagcagccgt ccccgcaccg gagcgctgcg
4621 tgccgcgcct cgccacatcc cggcggcaag ccgcgggatg cgcgccactg ccgtccgccc
4681 acaccggttc gcggtacgcg cgccacgcgc ccgagcgcac gctgctgtac gcgttggtag
4741 aggcgcacta cccggacttc attgcacgga tcgaagcgga gggccgctcg ctgcccgggt
4801 atgtccgcga ggcgttcgat gcctacctgc gttgcggcgt actcgagcac ggcttcctgc
4861 gggtggtgtg cgagcactgc cgtgcagaga ggctggtggc cttctcctgc aagaagcgcg
4921 ggttctgccc gagttgcggc gcgcgacgca tggccgagag tgcgcggcac ctggtcgagg
4981 aggtgttcgg cccgcggcct gtgcggcaat gggtgctgag ctttccgtac cccttgcgtt
5041 tcctgttcgc cagcaagcca gaagccattg gcccggtgct gggcatcgtg cagcgcgtga
5101 tcgccggctg gttggccgat caagccggca tcgaccgcgc cagcgcccag tgcggcgcgg
5161 tgacgctgat ccagcgtttc ggcagcgcgc tgaacctgaa catccacttc cacatgctgt
5221 ggctcgacgg cgtgtacgtg gaagccaccg agctgccgcg gcgcgaactg cgcctgcacc
5281 gcgcccgtgc gcccaccacc gcgcagttga cccagctggc agctaccatc gcgcaccggg
5341 tgtgtcggca cctgacgcgc aaaggctggc tcgaaggggа gggcgaatcg gccttcctgg
5401 cagacagcgc tgcaggcgac gacagcatgg atgggctgcg gatgagttcg atcacctacc
5461 gcatcgccac cggccgcgac gctggctgca aggtcgtcac gctgcaaacg ctgcccggtg
5521 acgccggttc gctggagggc gaagccggca aggtcggcgg cttctcactg catgccggcg
5581 tggcggccga agcacacgaa agccacaagc gggaaaagct gtgccgctac atcacgcgcc
5641 cggcgatcag cgagaagcgg ctgtcgatag cgctccaggg cagggtgcgt taccagctca
5701 agaccccgtg gcgcaatggc accacgcatg tggaatggga tccggtggat ttcatcgcca
5761 agctggcggc gctggtcccg ccacctcgcg cgcatctcac ccgcttccac ggcgtattcg
5821 ccccgaatgc aaacctgcgt gcgcagctga cgccctcggg gcgcggcaag cggcctgcgg
5881 gcgatgcggc gccagtggac gtcagcgccc acgacgcgcc gcgcagcccc gaggagaagc
5941 gccgtgcgat gagctgggcg caacggctca agcgggtctt ttccatcgac gtcaccgcct
6001 gcgtccactg cggtggcacc gtgcggatcg tcgccagcat cgaggaactc accgccatcc
6061 gcgccatcct cgcccacttc gagaagcacg gcgcgcggga agaagcgcac tacaggcccg
6121 cagcgcgcgc gccgccagtg caagccgcgt gacgatctgc cggctgcaca gccgacggcg
6181 aaaccggaat ccgagccgat gcggccacga tccgcagggc ggcgctcggc ccgctgtcgg
6241 gaatcagcga agcatggctg ctgacaacgc cgctgcgtgg ccccgcgatg ccgaaatccc
6301 actcacagac gtccgatccg tgcccaaaac ggggcttgcg cgaccgccgc ctacccagca
6361 gactgcccga aaagggcgtt tgaacttcct atacgcaacc cctcgcgcat gtcgcaccgc
6421 cgcctgaaag agaccatcgg cgtgcgctac aacgatttcg gcgtggtcga gcccatcgtc
6481 gccgacatcc gcgccatgct ggccacgcac gacggcatcg acaccacgca gacgctgatc
6541 gtgaacttca acgcgttcgg gccgagctcg ctggacatca tggtctacac cttcaccaag
6601 accacggtgt gggtcacctt ccacgagatc aagcaggatg tgctgctgcg catcgggcgc
6661 atcgtcgagt cgcatggcgc cgagatcgcg tttccgacgc agaccgtcta cctcgcgcag
6721 ccagaagagc cgcctgaggc catgcgacct gcagccgccc cgccagcagc gcgctgaatc
6781 cagcgcggtc cggacgcgag cgggtcaacc agatgcgagc gctgggcgct tgcgcagcag
6841 gcgcgccacc agcccagcgg ccaccagcac cagcaccgcg ccggcccagg ccgtggcctg
6901 cagcgcatcg gtgaagcccg cgcgtgcggc cgccagcaag gccgcgccct gccacgccgg

```
     6961 cagggtgtcg gccaggtgca cggcgccccc gagcgaggca ccggccgtct gcagcgcatc
     7021 ggccggcagg ccgggcagcg ccgcgctggt cagcgcctgc cggtagacca ccaggccgac
     7081 gctgccgaac agcgcgatgc ccagcgcgcc gctgaattcg gacaccgtct ccgacaaggc
     7141 cgaggccgcg cccgcgcgct cggacggcgc gctggtgatg atgatctcgt tgccgatggt
     7201 gaacaccggc gccaggccca ggcccttgac gatcgtggcc ggcaccagcc accacaggcc
     7261 ctgccccagc cccagcacgg cgaagccgaa cgccgctgcc gacaggccca cgacgaggat
     7321 gcgcgccgcc ggccagcgcg ccgcgagctg cggcgacaac agcgaaccga tgacgaagca
     7381 cagggagcag ggcagcgtgg ccagcccggc ctgcagcggc gacagcccca gcacgagctg
     7441 caggtactgc gtcatgaaga tgtagacgcc gaacatggcc agcgcggcca gcgcatacgc
     7501 cgccagcgcc gcgcggaaca gcgcgtgcgc gaacagccgc aggtccagca gcgggtaggc
     7561 gatgtggccc tggcggcgca ggaacagcgc cccgaccgcc agcccggcca gcagcgcagc
     7621 catcgaggcg aggccctctc catgctcggc caactgcttg agcccgtaga tcgtcagcag
     7681 caccgccgcc agcgacagca gcacgctggc caggtccagg tgccccgcgt ccggatcacg
     7741 atactcgggc aggaagcgag ggccgagcgc cagcgtcagc agcatcaccg gcacgttgag
     7801 ccagaacacg gcgccccagt ggaagaactc cagcaacacg ccgccgacca gcggaccgat
     7861 cgcgctgccc agcgaaaacg cggcgatcca cacgccgatg gcgaactggc gctggcgcgg
     7921 gtcgtggaac atgttgcgga ccagcgccat ggtggacggc gcgatggtgg cgccggccag
     7981 gccgagcaag gcgcgcgccg cgatcaacag cgcggcggta tcggccagcg ccgcgagcac
     8041 cgaggcgaat gcgaagaacg ccgcgccgat caacaacagc cggcgccggc cgatgcggtc
     8101 gcccagcgtg cccatggtga tcaggaagcc ggcgacgaag aagccgtaga tgtccaggat
     8161 ccagagaagc tgggcgctgg agggctgcag ttcacggctg agcaccggca gcgccaggtt
     8221 cagcaccgtg aggtccatgg cgtagaccag gcacggcagg gcgaccacgg ccaggccgat
     8281 ccattcgcgg cgggtggcct tccgacgatc cgctgcggtg tcgtggagcg tggcggacat
     8341 actcaaaact tccgggacaa gaatgcttta acgacgaagg caacgcgcgc atttcgacat
     8401 gccgtcgatg gcaacaccca tccgcattca ggcgcgcgtg acacgcatgc ctgttgcccg
     8461 tcgcacatca gggggcacgg tcgtagccgg ccagcagctg aagtactgac ctcatttttt
     8521 ccttcattca tttcaaacct ctttttaatg ttttaggtta tcgccagctc ggcaacttga
     8581 tactctggtg aagcccaact ttgttttagg gcgactgccc tgctgcgtaa catcgttgct
     8641 gctccataac atcaaacatc gacccacggc gtaacgcgct tgctgcttgg atgcccgagg
     8701 catagactgt acaaaaaaac agtcataaca agccatgaaa accgccactg cgccgttacc
     8761 accgctgcgt tcggtcaagg ttctggacca gttgcgtgag cgcatacgct acttgcatta
     8821 cagtttacga accgaacagg cttatgtcca ctgggttcgt gccttcatcc gtttccacgg
     8881 tgtgcgtcac ccggcaacct tgggcagcag cgaagtcgag gcatttctgt cctggctggc
     8941 gaacgagcgc aaggtttcgg tctccacgca tcgtcaggca ttggcggcct tgctgttctt
     9001 ctacggcaag gtgctgtgca cggatctgcc ctggcttcag gagatcggaa gacctcggcc
     9061 gtcgcggcgc ttgccggtgg tgctgacccc ggatgaagtg gttcgcatcc tcggttttct
     9121 ggaaggcgag catcgtttgt tcgcccagct tctgtatgga acgggcatgc ggatcagtga
     9181 gggtttgcaa ctgcgggtca aggatctgga tttcgatcac ggcacgatca tcgtgcggga
     9241 gggcaagggc tccaaggatc gggccttgat gttacccgag agcttggcac ccagcctgcg
     9301 cgagcagctg tcgcgtgcac gggcatggtg gctgaaggac caggccgagg gccgcagcgg
     9361 cgttgcgctt cccgacgccc ttgagcggaa gtatccgcgc gccgggcatt cctggcggca
     9421 ctgttgcaaa tagtcggtgg tgataaactt atcatcccct tttgctgatg gagctgcaca
     9481 tgaacccatt caaaggccgg cattttcagc gtgacatcat tctgtgggcc gtacgctggt
     9541 actgcaaata cggcatcagt taccgtgagc tgcaggagat gctggctgaa cgcggagtga
     9601 atgtcgatca ctccacgatt taccgctggg ttcagcgtta tgcgcctgaa atggaaaaac
     9661 ggctgcgctg gtactggcgt aacccttccg atctttgccc gtggcacatg gatgaaacct
     9721 acgtgaaggt caatggccgc tgggcgtatc tgtaccgggc cgtcgacagc cggggccgca
     9781 ctgtcgattt ttatctctcc tcccgtcgta acagcaaagc tgcataccgg tttctgggta
     9841 aaatcctcaa caacgtgaag aagtggcaga tcccgcgatt catcaacacg gataaagcgc
     9901 ccgcctatgg tcgcgcgctt gctctgctca aacgcgaagg ccggtgcccg tctgacgttg
     9961 aacaccgaca gattaagtac cggaacaacg tgattgaatg cgatcatggc aaactgaaac
    10021 ggataatcgg cgccacgctg ggatttaaat ccatgaagac ggcttacgcc accatcaaag
    10081 gtattgaggt gatgcgtgca ctacgcaaag gccaggcctc agcattttat tatggtgatc
    10141 ccctgggcga aatgcgcctg gtaagcagag tttttgaaat gtaaggcctt tgaataagac
    10201 aaaaggctgc ctcatcgcta actttgcaac agtgcc
//

LOCUS       KR259131               10483 bp    DNA     circular BCT 08-JUL-2015
DEFINITION  Escherichia coli strain EC3587 plasmid pEC3587, complete sequence.
ACCESSION   KR259131
VERSION     KR259131
```

```
KEYWORDS    .
SOURCE      Escherichia coli
  ORGANISM  Escherichia coli
            Bacteria; Proteobacteria; Gammaproteobacteria; Enterobacteriales;
            Enterobacteriaceae; Escherichia.
REFERENCE   1  (bases 1 to 10483)
  AUTHORS   Wang,D., Huang,X. and Chen,J.
  TITLE     A plasmid from the isolate EC3587 of Escherichia coli involving
            relevant genes haboring 16S rRNA methylases,blaTEM-12 and novel
            qepA3 genes from patient's sputum in Taizhou municipal hospital of
            China
  JOURNAL   Unpublished
REFERENCE   2  (bases 1 to 10483)
  AUTHORS   Wang,D., Huang,X. and Chen,J.
  TITLE     Direct Submission
  JOURNAL   Submitted (22-APR-2015) Department of Clinical Lab Medicine,
            Taizhou Municipal Hospital and Institute of Molecular Diagnostics
            of Taizhou University, 381 East Rd. of Zhongshan in Jiaojiang of
            Taizhou, Taizhou, Zhejiang 318000, China
COMMENT     ##Assembly-Data-START##
            Sequencing Technology :: Sanger dideoxy sequencing
            ##Assembly-Data-END##
FEATURES             Location/Qualifiers
     source          1..10483
                     /organism="Escherichia coli"
                     /mol_type="genomic DNA"
                     /strain="EC3587"
                     /host="Homo sapiens"
                     /db_xref="taxon:562"
                     /plasmid="pEC3587"
                     /country="China: Taizhou municipal hospital of Zhejiang
                     province"
                     /lat_lon="28 N 122 E"
                     /collection_date="17-Aug-2012"
                     /collected_by="Dongguo Wang"
                     /note="genotype:
                     tnpA;tnpR;blaTEM-12;rmtB;tnpA;qepA3;dfr2;intI1-groEL;tnpA"
     gene            52..768
                     /gene="tnpA"
                     /note="insertion sequence:IS26; tnpA gene; transposase"
                     /pseudo
     gene            1037..1630
                     /gene="tnpR"
                     /pseudo
     gene            1813..2673
                     /gene="blaTEM-12"
     CDS             1813..2673
                     /gene="blaTEM-12"
                     /note="beta-lactamase"
                     /codon_start=1
                     /transl_table=11
                     /product="TEM-12"
                     /protein_id="AKP49179"
                     /translation="MSIQHFRVALIPFFAAFCLPVFAHPETLVKVKDAEDQLGARVGY
                     IELDLNSGKILESFRPEERFPMMSTFKVLLCGAVLSRVDAGQEQLGRRIHYSQNDLVE
                     YSPVTEKHLTDGMTVRELCSAAITMSDNTAANLLLTTIGGPKELTAFLHNMGDHVTRL
                     DSWEPELNEAIPNDERDTTMPAAMATTLRKLLTGELLTLASRQQLIDWMEADKVAGPL
                     LRSALPAGWFIADKSGAGERGSRGIIAALGPDGKPSRIVVIYTTGSQATMDERNRQIA
                     EIGASLIKHW"
     gene            2843..3598
                     /gene="rmtB"
```

```
     CDS             2843..3598
                     /gene="rmtB"
                     /codon_start=1
                     /transl_table=11
                     /product="16S rRNA methylase"
                     /protein_id="AKP49180"
                     /translation="MNINDALTSILASKKYRALCPDTVRRILTEEWGRHKSPKQTVEA
                     ARTRLHGICGAYVTPESLKAAAAALSAGDVKKALSLHASTKERLAELDTLYDFIFSAE
                     TPRRVLDIACGLNPLALYERGIASVWGCDIHQGLGDVITPFAREKDWDFTFALQDVLC
                     APPAEAGDLALIFKLLPLLEREQAGSAMALLQSLNTPRMAVSFPTRSLGGRGKGMEAN
                     YAAWFEGGLPAEFEIEDKKTIGTELIYLIKKNG"
     gene            5197..6399
                     /gene="tnpA"
                     /note="insertion sequence:ISCR3; tnpA gene; transposase
                     ISCR3C"
                     /pseudo
     gene            complement(7052..8587)
                     /gene="qepA3"
     CDS             complement(7052..8587)
                     /gene="qepA3"
                     /note="quinolone efflux pump"
                     /codon_start=1
                     /transl_table=11
                     /product="QepA3"
                     /protein_id="AKP49181"
                     /translation="MSATLHDTAADRRKATRREWIGLAVVALPCLVYAMDLTVLNLAL
                     PVLSRELQPSSAQLLWILDIYGFFVAGFLITMGTLGDRIGRRRLLLIGAAFFAFASVL
                     AALADTAALLIAARALLGLAGATIAPSTMALVRNMFHDPRQRQFAIGVWIAAFSLGSA
                     IGPLVGGVLLEFFHWGAVFWLNVPVMLLTLALGPRFLPEYRDPDAGHLDLASVLLSLA
                     AVLLTIYGLKQLAEHGEGLASMAALLAGLAVGALFLRRQGHIAYPLLDLRLFAHALFR
                     AALAAYALAALAMFGVYIFMTQYLQLVLGLSPLQAGLATLPCSLCFVIGSLLSPQLAA
                     RWPAARILVVGLSAAAFGFAVLGLGQGLWWLVPATIVKGLGLAPVFTIGNEIIITSAP
                     SERAGAASALSETVSEFSGALGIALFGSVGLVVYRQALTSAALPGLPADALQTAGASL
                     GGAVHLADTLPAWQGAALLAAARAGFTDALQATAWAGAVLVLVAAGLVARLLRKRPAL
                     ASG"
     gene            8646..8756
                     /gene="dfr2"
                     /note="dfr2 gene; Dfr2"
                     /pseudo
     gene            8982..9680
                     /gene="integrase fusion groEL/intI1"
                     /note="similar to chaperonin/integrase fusion protein;
                     integrase fusion gene groEL/intI1; integrase fusion
                     protein groEL/intI1"
                     /pseudo
     gene            9715..10431
                     /gene="tnpA"
                     /note="tnpA gene; transposase"
                     /pseudo
ORIGIN
        1 ggcactgttg caaatagtcg gtggtgataa acttatcatc cccttttgct gatggagctg
       61 cacatgaacc cattcaaagg ccggcatttt cagcgtgaca tcattctgtg ggccgtacgc
      121 tggtactgca aatacggcat cagttaccgt gagctgcagg agatgctggc tgaacgcgga
      181 gtgaatgtcg atcactccac gatttaccgc tgggttcagc gttatgcgcc tgaaatggaa
      241 aaacggctgc gctggtactg gcgtaaccct tccgatcttt gcccgtggca catggatgaa
      301 acctacgtga aggtcaatgg ccgctgggcg tatctgtacc gggccgtcga cagccggggc
      361 cgcactgtcg atttttatct ctcctcccgt cgtaacagca aagctgcata ccggtttctg
      421 ggtaaaatcc tcaacaacgt gaagaagtgg cagatcccgc gattcatcaa cacggataaa
      481 gcgcccgcct atggtcgcgc gcttgctctg ctcaaacgcg aaggccggtg cccgtctgac
      541 gttgaacacc gacagattaa gtaccggaac aacgtgattg aatgcgatca tggcaaactg
      601 aaacggataa tcggcgccac gctgggattt aaatccatga agacggctta cgccaccatc
```

661 aaaggtattg aggtgatgcg tgcactacgc aaaggccagg cctcagcatt ttattatggt
721 gatcccctgg gcgaaatgcg cctggtaagc agagtttttg aaatgtaagg cctttgaata
781 agacaaaagg ctgcctcatc gctaactttg caacagtgcc aaatatcgtg ccagctgaag
841 ctcatccggt tcaccggtga atctgccata gctttcagtc tgctcagtgg tcagaaagtc
901 aacgggcata tcggcctccc tgcctgacgg gcatttagta acatttttcc aaccgtacga
961 aatgttataa attatcggac atcgtaaaac tgttacatta atatgtctat taaatcgtaa
1021 atttgtaata atagacatga gttgtccgat attcgattta aggtacattt ttatgcgact
1081 ttttggttac gctcgggtct caaccagtca gcagtctctt gatcttcagg tcagagcact
1141 caaagacgca ggtgtgaaag caaaccgtat atttaccgat aaggcatccg gcagttcaac
1201 agaccgggaa gggctggatt tgctgaggat gaaggtggag gaaggtgatg tcattctggt
1261 taagaagctc gaccgtcttg gccgcgacac tgccgatatg atccaactga taaaggaatt
1321 tgacgctcag ggcgtggcag tccggttcat tgatgacggg atcagtaccg acggtgatat
1381 ggggcaaatg gtggtcacca tcctgtcggc tgtggcacag gctgaacgcc ggaggatcct
1441 agaacgcacg aatgagggcc gacaggaagc aaagctgaaa ggaatcaaat ttggccgcag
1501 gcgtaccgtg gacaggaacg tcgtgctgac gcttcatcag aagggcactg gtgcaacgga
1561 aattgctcat cagctcagta ttgcccgctc cacggtttat aaaattcttg aagacgaaag
1621 ggcctcgtga tacgcctatt tttataggtt aatgtcatga taataatggt ttcttagacg
1681 tcaggtggca cttttcgggg aaatgtgcgc ggaaccccta tttgtttatt tttctaaata
1741 cattcaaata tgtatccgct catgagacaa taaccctggt aaatgcttca ataatattga
1801 aaaaggaaga gtatgagtat tcaacatttc cgtgtcgccc ttattccctt ttttgcggca
1861 ttttgccttc ctgtttttgc tcacccagaa acgctggtga aagtaaaaga tgctgaagat
1921 cagttgggtg cacgagtggg ttacatcgag ctggatctca acagcggtaa gatccttgag
1981 agttttcgcc ccgaagaacg ttttccaatg atgagcactt ttaaagttct gctatgtggt
2041 gcggtattat cccgtgttga cgccgggcaa gagcaactcg gtcgccgcat acactattct
2101 cagaatgact tggttgagta ctcaccagtc acagaaaagc atcttacgga tggcatgaca
2161 gtaagagaat tatgcagtgc tgccataacc atgagtgata acactgcggc caacttactt
2221 ctgacaacga tcggaggacc gaaggagcta accgcttttt tgcacaacat gggggatcat
2281 gtaacccgcc ttgatagttg ggaaccggag ctgaatgaag ccataccaaa cgacgagcgt
2341 gacaccacga tgcctgcagc aatggcaaca acgttgcgca aactattaac tggcgaacta
2401 cttactctag cttcccggca acaattaata gactggatgg aggcggataa agttgcagga
2461 ccacttctgc gctcggccct tccggctggc tggtttattg ctgataaatc tggagccggt
2521 gagcgtggat ctcgcggtat cattgcagca ctggggccag atggtaagcc ctcccgtatc
2581 gtagttatct acacgacggg gagtcaggca actatggatg aacgaaatag acagatcgct
2641 gagataggtg cctcactgat taagcattgg taactgtcag accaagttta ctcatatata
2701 ctttagattg atttaaaact tcatttttaa tttaaaagga tctaggtgaa gatccttttt
2761 gataatctca tgaccaaaat cccttaacgt gagttttcgt tccactgagc gtcagacccc
2821 gaaaacgatt taggagacac cgatgaacat caacgatgcc ctcacctcca tcctggcctc
2881 aaaaaaatac cgcgcccttt gcccggatac cgtgcggcgc atcctgactg aggaatgggg
2941 gcggcataaa tcccccaaac agaccgtaga ggctgcacgc acccggctgc atggaatttg
3001 cggggcatat gtcaccccgg aatcgctcaa ggctgctgcc gccgcgcttt ctgcgggcga
3061 tgtaaaaaag gcattgtcgc tgcatgcctc caccaaggag cgactggccg agctggatac
3121 cctgtacgat tttatctttt cagccgaaac tccccgccgc gtgctggata tcgcctgcgg
3181 tcttaacccc ttggcgctat acgagcgcgg cattgcatcc gtgtggggct gtgatatcca
3241 ccagggattg gggatgtca tcaccccctt tgctagggaa aaagattggg attttacctt
3301 tgccctgcag gatgtgctgt gtgcgccgcc cgccgaagcc ggcgacctgg cgctgatttt
3361 taagcttttg cccctgctgg agcgggagca ggccggttct gccatggcac ttttacaatc
3421 cctcaatacc ccgcgcatgg ctgtcagctt tcccacgcgt agtttaggcg ggcgtggaaa
3481 aggcatggag gcgaactacg ccgcatggtt cgagggcggc ttgcccgccg agtttgagat
3541 tgaggataaa aagaccatcg gaacagaact tatatacttg ataaaaaaga atggataagc
3601 caatcagaag gcaaatcatc taaaaaagag gaacgaaaaa tatttttcgt tcctcttttg
3661 ttctatcgaa caagcacact acgactgctg tttttgaagc agccttttat agctcagctc
3721 gatgcccaac gcccccagcg gcgctgtaag tataatggca agaaccgcca ccgcaagtat
3781 ggtttcccca ctgccaagcc ccatcgccag cggaatagcg ccgattgcag cctgcaccgt
3841 tgccttgggc agataggcaa tcatgcagaa caggcgctcc ttccgggata ggtctgtacc
3901 cagcgtactc atccacacgc ccagcatgcg aaacagcagc gcagctgtga tcagcagcat
3961 gccgcttagc ccggcggaga aaaggtaacg gatgttgacc gttgcaccca ccagcacaaa
4021 cagccagatt tctgccgcca cccaaagctt tgaaaatttg ccggatatac gctttgcaac
4081 cggggcgttg gtttttaaaa gggtgacgcc catccccatc accgccagca atccggaaaa
4141 tagcgccctt tcgcccaggg ttttctccaa cgcaaccagc gcaaaggatg cgcccagcag
4201 caggagcacc ttgatcgtat cacgcatatg tacccgctta aaaagggctg ccggccggca
4261 aggtgctgtg cacggatctg ccctggcagg gcatcaacga agaccagaac ctgggcatcg

4321 ccatcacccg ccgtgcgctg gaagccccgc tgcgcgccat cgtggccaac gccggtgaag
4381 aaccgagcgt gatcgtggcc aacgtcaagg ccggcgaagg cagctacggc tacaacgccg
4441 ccaccggcga gttcggcgac atgatcgcca tgggcatcct ggacccgacc aaggtgaccc
4501 gctcggccct gcagcacgcc gcttccgtcg ccggccttgc gatcacgacc gaagtggtcg
4561 tggccgaagt gccgaagaag gaagagccgg ccatgccggg tgctggcggt atgggcggca
4621 tgggcggcat ggatttctga tccggttggc ccggtcgtca gggaaccgga ccgcgccagc
4681 gcggtccgat cccggcaacg acccgacatc aaggccccaa ggacggggcc ggagcccggc
4741 agcgatgccg ggctttttgt tgtgcccgcg ccgcggcaat gtctgacgcg aagatcagaa
4801 cgcaccgata cgaacgtgcg aacacaggcg caacactgag cagccgtccc cgcaccggag
4861 cgctgcgtgc cgcgcctcgc cacatcccgg cggcaagccg cgggatgcgc gccactgccg
4921 tccgcccaca ccggttcgcg gtacgcgcgc cacgcgcccg agcgcacgct gctgtacgcg
4981 ttggtagagg cgcactaccc ggacttcatt gcacggatcg aagcggaggg ccgctcgctg
5041 cccgggtatg tccgcgaggc gttcgatgcc tacctgcgtt gcggcgtact cgagcacggc
5101 ttcctgcggg tggtgtgcga gcactgccgt gcagagaggc tggtggcctt ctcctgcaag
5161 aagcgcgggt tctgcccgag ttgcggcgcg cgacgcatgg ccgagagtgc gcggcacctg
5221 gtcgaggagg tgttcggccc gcggcctgtg cggcaatggg tgctgagctt tccgtacccc
5281 ttgcgtttcc tgttcgccag caagccagaa gccattggcc cggtgctggg catcgtgcag
5341 cgcgtgatcg ccggctggtt ggccgatcaa gccggcatcg accgcgccag cgcccagtgc
5401 ggcgcggtga cgctgatcca gcgtttcggc agcgcgctga acctgaacat ccacttccac
5461 atgctgtggc tcgacggcgt gtacgtggaa gccaccgagc tgccgcggcg cgaactgcgc
5521 ctgcaccgcg cccgtgcgcc caccaccgcg cagttgaccc agctggcagc taccatcgcg
5581 caccgggtgt gtcggcacct gacgcgcaaa ggctggctcg aaggggaggg cgaatcggcc
5641 ttcctggcag acagcgctgc aggcgacgac agcatggatg ggctgcggat gagttcgatc
5701 acctaccgca tcgccaccgg ccgcgacgct ggctgcaagg tcgtcacgct gcaaacgctg
5761 cccggtgacg ccggttcgct ggagggcgaa gccggcaagg tcggcggctt ctcactgcat
5821 gccggcgtgg cggccgaagc acacgaaagc cacaagcggg aaaagctgtg ccgctacatc
5881 acgcgcccgg cgatcagcga gaagcggctg tcgatagcgc tccagggcag ggtgcgttac
5941 cagctcaaga ccccgtggcg caatggcacc acgcatgtgg aatgggatcc ggtggatttc
6001 atcgccaagc tggcggcgct ggtcccgcca cctcgcgcgc atctcacccg cttccacggc
6061 gtattcgccc cgaatgcaaa cctgcgtgcg cagctgacgc cctcggggcg cggcaagcgg
6121 cctgcgggcg atgcggcgcc agtggacgtc agcgcccacg acgcgccgcg cagccccgag
6181 gagaagcgcc gtgcgatgag ctgggcgcaa cggctcaagc gggtcttttc catcgacgtc
6241 accgcctgcg tccactgcgg tggcaccgtg cggatcgtcg ccagcatcga ggaacccacc
6301 gccatccgcg ccatcctcgc ccacttcgag aagcacggcg cgcgggaaga agcgcactac
6361 aggcccgcag cgcgcgcgcc gccagtgcaa gccgcgtgac gatctgccgg ctgcacagcc
6421 gacggcgaaa ccggaatccg agccgatgcg gccacgatcc gcagggcggc gctcggcccg
6481 ctgtcgggaa tcagcgaagc atggctgctg acaacgccgc tgcgtggccc cgcgatgccg
6541 aaatcccact cacagacgtc cgatccgtgc ccaaaacggg gcttgcgcga ccgccgccta
6601 cccagcagac tgcccgaaaa gggcgtttga acttcctata cgcaacccct cgcgcatgtc
6661 gcaccgccgc ctgaaagaga ccatcggcgt gcgctacaac gatttcggcg tggtcgagcc
6721 catcgtcgcc gacatccgcg ccatgctggc cacgcacgac ggcatcgaca ccacgcagac
6781 gctgatcgtg aacttcaacg cgttcgggcc gagctcgctg gacatcatgg tctacacctt
6841 caccaagacc acggtgtggg tcaccttcca cgagatcaag caggatgtgc tgctgcgcat
6901 cgggcgcatc gtcgagtcgc atggcgccga gatcgcgttt ccgacgcaga ccgtctacct
6961 cgcgcagcca gaagagccgc ctgaggccat gcgacctgca gccgccccgc cagcagcgcg
7021 ctgaatccag cgcggtccgg acgcgagcgg gtcaaccaga tgcgagcgct gggcgcttgc
7081 gcagcaggcg cgccaccagc ccagcggcca ccagcaccag caccgcgccg gcccaggccg
7141 tggcctgcag cgcatcggtg aagcccgcgc gtgcggccgc cagcaaggcc gcgccctgcc
7201 acgccggcag ggtgtcggcc aggtgcacgg cgccccccgag cgaggcaccg gccgtctgca
7261 gcgcatcggc cggcaggccg ggcagcgccg cgctggtcag cgcctgccgg tagaccacca
7321 ggccgacgct gccgaacagc gcgatgccca gcgcgccgct gaattcggac accgtctccg
7381 acaaggccga ggccgcgccc gcgcgctcgg acggcgcgct ggtgatgatg atctcgttgc
7441 cgatggtgaa caccggcgcc aggcccaggc ccttgacgat cgtggccggc accagccacc
7501 acaggccctg ccccagcccc agcacggcga agccgaacgc cgctgccgac aggcccacga
7561 cgaggatgcg cgccgccggc cagcgcgccg cgagctgcgg cgacaacagc gaaccgatga
7621 cgaagcacag ggagcagggc agcgtggcca gcccggcctg cagcggcgac agccccagca
7681 cgagctgcag gtactgcgtc atgaagatgt agacgccgaa catggccagc gcggccagcg
7741 catacgccgc cagcgccgcg cggaacagcg cgtgcgcgaa cagccgcagg tccagcagcg
7801 ggtaggcgat gtggccctgg cggcgcagga acagcgcccc gaccgccagc ccggccagca
7861 gcgcagccat cgaggcgagg ccctctccat gctcggccaa ctgcttgagc ccgtagatcg
7921 tcagcagcac cgccgccagc gacagcagca cgctggccag gtccaggtgc cccgcgtccg

```
     7981 gatcacgata ctcgggcagg aagcgagggc cgagcgccag cgtcagcagc atcaccggca
     8041 cgttgagcca gaacacggcg ccccagtgga agaactccag caacacgccg ccgaccagcg
     8101 gaccgatcgc gctgcccagc gaaaacgcgg cgatccacac gccgatggcg aactggcgct
     8161 ggcgcgggtc gtggaacatg ttgcggacca gcgccatggt ggacggcgcg atggtggcgc
     8221 cggccaggcc gagcaaggcg cgcgccgcga tcaacagcgc ggcggtatcg gccagcgccg
     8281 cgagcaccga ggcgaatgcg aagaacgccg cgccgatcaa caacagccgg cgccggccga
     8341 tgcggtcgcc cagcgtgccc atggtgatca ggaagccggc gacgaagaag ccgtagatgt
     8401 ccaggatcca gagaagctgg gcgctggagg gctgcagttc acggctgagc accggcagcg
     8461 ccaggttcag caccgtgagg tccatggcgt agaccaggca cggcagggcg accacggcca
     8521 ggccgatcca ttcgcggcgg gtggccttcc gacgatccgc tgcggtgtcg tggagcgtgg
     8581 cggacatact caaaacttcc gggacaagaa tgctttaacg acgaaggcaa cgcgcgcatt
     8641 tcgacatgcc gtcgatggca acacccatcc gcattcaggc gcgcgtgaca cgcatgcctg
     8701 ttgcccgtcg cacatcaggg ggcacggtcg tagccggcca gcagctgaag tactgacctc
     8761 attttttcct tcattcattt caaacctctt tttaatgttt taggttatcg ccagctcggc
     8821 aacttgatac tctggtgaag cccaactttg ttttagggcg actgccctgc tgcgtaacat
     8881 cgttgctgct ccataacatc aaacatcgac ccacggcgta acgcgcttgc tgcttggatg
     8941 cccgaggcat agactgtaca aaaaaacagt cataacaagc catgaaaacc gccactgcgc
     9001 cgttaccacc gctgcgttcg gtcaaggttc tggaccagtt gcgtgagcgc atacgctact
     9061 tgcattacag cttaccaacc gaacaggctt atgtccactg ggttcgtgcc ttcatccgtt
     9121 tccacggtgt gcgtcacccg gcaaccttgg gcagcagcga agtcgaggca tttctgtcct
     9181 ggctggcgaa cgagcgcaag gtttcggtct ccacgcatcg tcaggcattg gcggccttgc
     9241 tgttcttcta cggcaaggtt ctgtgcacgg atctgccctg gcttcaggag atcggaagac
     9301 ctcggccgtc gcggcgcttg ccggtggtgc tgaccccgga tgaagtggtt cgcatcctcg
     9361 gttttctgga aggcgagcat cgtttgttcg cccagcttct gtatggaacg ggcatgcgga
     9421 tcagtgaggg tttgcaactg cgggtcaagg atctggattt cgatcacggc acgatcatcg
     9481 tgcgggaggg caagggctcc aaggatcggg ccttgatgtt acccgagagc ttggcaccca
     9541 gcctgcgcga gcagctgtcg cgtgcacggg catggtggct gaaggaccag gccgagggcc
     9601 gcagcggcgt tgcgcttccc gacgcccttg agcggaagta tccgcgcgcc gggcattcct
     9661 ggcggcactg ttgcaaatag tcggtggtga taaacttatc atccccttttt gctgatggag
     9721 ctgcacatga acccattcaa aggccggcat tttcagcgtg acatcattct gtgggccgta
     9781 cgctggtact gcaaatacgg catcagttac cgtgagctgc aggagatgct ggctgaacgc
     9841 ggagtgaatg tcgatcactc cacgatttac cgctgggttc agcgttatgc gcctgaaatg
     9901 gaaaaacggc tgcgctggta ctggcgtaac ccttccgatc tttgcccgtg gcacatggat
     9961 gaaacctacg tgaaggtcaa tggccgctgg gcgtatctgt accgggccgt cgacagccgg
    10021 ggccgcactg tcgattttta tctctcctcc cgtcgtaaca gcaaagctgc ataccggttt
    10081 ctgggtaaaa tcctcaacaa cgtgaagaag tggcagatcc cgcgattcat caacacggat
    10141 aaagcgcccg cctatggtcg cgcgcttgct ctgctcaaac gcgaaggccg gtgcccgtct
    10201 gacgttgaac accgacagat taagtaccgg aacaacgtga ttgaatgcga tcatggcaaa
    10261 ctgaaacgga taatcggcgc cacgctggga tttaaatcca tgaagacggc ttacgccacc
    10321 atcaaaggta ttgaggtgat gcgtgcacta cgcaaaggcc aggcctcagc attttattat
    10381 ggtgatcccc tgggcgaaat gcgcctggta agcagagttt ttgaaatgta aggcctttga
    10441 ataagacaaa aggctgcctc atcgctaact ttgcaacagt gcc
//

LOCUS       KR259132               10359 bp    DNA     circular BCT 08-JUL-2015
DEFINITION  Citrobacter koseri strain CD4359 plasmid pCD4359, complete
            sequence.
ACCESSION   KR259132
VERSION     KR259132
KEYWORDS    .
SOURCE      Citrobacter koseri
  ORGANISM  Citrobacter koseri
            Bacteria; Proteobacteria; Gammaproteobacteria; Enterobacteriales;
            Enterobacteriaceae; Citrobacter.
REFERENCE   1  (bases 1 to 10359)
  AUTHORS   Wang,D.
  TITLE     A plasmid from the isolate CD4359 of Citrobacter diversus haboring
            16S rRNA methylases, blaTEM-1 and novel qepA3 genes from patient's
            sputum in Taizhou municipal hospital of China
  JOURNAL   Unpublished
REFERENCE   2  (bases 1 to 10359)
```

```
  AUTHORS   Wang,D.
  TITLE     Direct Submission
  JOURNAL   Submitted (22-APR-2015) Department of Clinical Lab Medicine,
            Taizhou Municipal Hospital and Institute of Molecular Diagnostics
            of Taizhou University, 381 East Rd. of Zhongshan in Jiaojiang of
            Taizhou, Taizhou, Zhejiang 318000, China
COMMENT     ##Assembly-Data-START##
            Sequencing Technology :: Sanger dideoxy sequencing
            ##Assembly-Data-END##
FEATURES             Location/Qualifiers
     source          1..10359
                     /organism="Citrobacter koseri"
                     /mol_type="genomic DNA"
                     /strain="CD4359"
                     /host="Homo sapiens"
                     /db_xref="taxon:545"
                     /plasmid="pCD4359"
                     /country="China: Taizhou municipal hospital of Zhejiang
                     province"
                     /lat_lon="28 N 122 E"
                     /collection_date="24-Sep-2011"
                     /collected_by="Dongguo Wang"
                     /note="genotype:
                     tnpA;tnpR'blaTEM-1;rmtB;intI1;tnpA;qepA3;intI1-groEL;tnpA"
     gene            52..768
                     /gene="tnpA"
                     /note="insertion sequence:IS26; tnpA gene; transposase
                     IS26"
                     /pseudo
     gene            1037..1630
                     /gene="tnpR"
                     /note="truncated resolvase; tnpR gene; TnpR"
                     /pseudo
     gene            1813..2673
                     /gene="blaTEM-1"
     CDS             1813..2673
                     /gene="blaTEM-1"
                     /note="beta-lactamase"
                     /codon_start=1
                     /transl_table=11
                     /product="TEM-1 protein"
                     /protein_id="AKP49182"
                     /translation="MSIQHFRVALIPFFAAFCLPVFAHPETLVKVKDAEDQLGARVGY
                     IELDLNSGKILESFRPEERFPMMSTFKVLLCGAVLSRVDAGQEQLGRRIHYSQNDLVE
                     YSPVTEKHLTDGMTVRELCSAAITMSDNTAANLLLTTIGGPKELTAFLHNMGDHVTRL
                     DRWEPELNEAIPNDERDTTMPAAMATTLRKLLTGELLTLASRQQLIDWMEADKVAGPL
                     LRSALPAGWFIADKSGAGERGSRGIIAALGPDGKPSRIVVIYTTGSQATMDERNRQIA
                     EIGASLIKHW"
     gene            2759..3514
                     /gene="rmtB"
     CDS             2759..3514
                     /gene="rmtB"
                     /codon_start=1
                     /transl_table=11
                     /product="16S rRNA methylase"
                     /protein_id="AKP49183"
                     /translation="MNINDALTSILASKKYRALCPDTVRRILTEEWGRHKSPKQTVEA
                     ARTRLHGICGAYVTPESLKAAAAALSAGDVKKALSLHASTKERLAELDTLYDFIFSAE
                     TPRRVLDIACGLNPLALYERGIASVWGCDIHQGLGDVITPFAREKDWDFTFALQDVLC
                     APPAEAGDLALIFKLLPLLEREQAGSAMALLQSLNTPRMAVSFPTRSLGGRGKGMEAN
                     YAAWFEGGLPAEFEIEDKKTIGTELIYLIKKNG"
```

```
     gene            3908..4699
                     /gene="intI1"
                     /note="intI1 gene; integrase"
                     /pseudo
     gene            5075..6277
                     /gene="tnpA"
                     /note="insertion sequence:ISCR3; tnpA gene; transposase
                     ISCR3C"
                     /pseudo
     gene            complement(6930..8465)
                     /gene="qepA3"
     CDS             complement(6930..8465)
                     /gene="qepA3"
                     /note="quinolone efflux pump"
                     /codon_start=1
                     /transl_table=11
                     /product="QepA3"
                     /protein_id="AKP49184"
                     /translation="MSATLHDTAADRRKATRREWIGLAVVALPCLVYAMDLTVLNLAL
                     PVLSRELQPSSAQLLWILDIYGFFVAGFLITMGTLGDRIGRRRLLLIGAAFFAFASVL
                     AALADTAALLIAARALLGLAGATIAPSTMALVRNMFHDPRQRQFAIGVWIAAFSLGSA
                     IGPLVGGVLLEFFHWGAVFWLNVPVMLLTLALGPRFLPEYRDPDAGHLDLASVLLSLA
                     AVLLTIYGLKQLAEHGEGLASMAALLAGLAVGALFLRRQGHIAYPLLDLRLFAHALFR
                     AALAAYALAALAMFGVYIFMTQYLQLVLGLSPLQAGLATLPCSLCFVIGSLLSPQLAA
                     RWPAARILVVGLSAAAFGFAVLGLGQGLWWLVPATIVKGLGLAPVFTIGNEIIITSAP
                     SERAGAASALSETVSEFSGALGIALFGSVGLVVYRQALTSAALPGLPADALQTAGASL
                     GGAVHLADTLPAWQGAALLAAARAGFTDALQATAWAGAVLVLVAAGLVARLLRKRPAL
                     ASG"
     gene            8858..9556
                     /gene="integrase fusion groEL/intI1"
                     /note="similar to chaperonin/integrase fusion protein;
                     integrase fusion gene groEL/intI1; integrase fusion
                     protein groEL/intI1"
                     /pseudo
     gene            9591..10307
                     /gene="tnpA"
                     /note="tnpA gene; transposase IS26"
                     /pseudo
ORIGIN
        1 ggcactgttg caaatagtcg gtggtgataa acttatcatc cccttttgct gatggagctg
       61 cacatgaacc cattcaaagg ccggcatttt cagcgtgaca tcattctgtg ggccgtacgc
      121 tggtactgca aatacggcat cagttaccgt gagctgcagg agatgctggc tgaacgcgga
      181 gtgaatgtcg atcactccac gatttaccgc tgggttcagc gttatgcgcc tgaaatggaa
      241 aaacggctgc gctggtactg gcgtaaccct tccgatcttt gcccgtggca catggatgaa
      301 acctacgtga aggtcaatgg ccgctgggcg tatctgtacc gggccgtcga cagccggggc
      361 cgcactgtcg atttttatct ctcctcccgt cgtaacagca aagctgcata ccggtttctg
      421 ggtaaaatcc tcaacaacgt gaagaagtgg cagatcccgc gattcatcaa cacggataaa
      481 gcgcccgcct atggtcgcgc gcttgctctg ctcaaacgcg aaggccggtg cccgtctgac
      541 gttgaacacc gacagattaa gtaccggaac aacgtgattg aatgcgatca tggcaaactg
      601 aaacggataa tcggcgccac gctgggattt aaatccatga agacggctta cgccaccatc
      661 aaaggtattg aggtgatgcg tgcactacgc aaaggccagg cctcagcatt ttattatggt
      721 gatcccctgg gcgaaatgcg cctggtaagc agagtttttg aaatgtaagg cctttgaata
      781 agacaaaagg ctgcctcatc gctaactttg caacagtgcc aaatatcgtg ccagctgaag
      841 ctcatccggt tcaccggtga atctgccata gctttcagtc tgctcagtgg tcagaaagtc
      901 aacgggcata tcggcctccc tgcctgacgg gcatttagta acatttttcc aaccgtacga
      961 aatgttataa attatcggac atcgtaaaac tgttacatta atatgtctat taaatcgtaa
     1021 atttgtaata atagacatga gttgtccgat attcgattta aggtacattt ttatgcgact
     1081 ttttggttac gctcgggtct caaccagtca gcagtctctt gatcttcagg tcagagcact
     1141 caaagacgca ggtgtgaaag caaaccgtat atttaccgat aaggcatccg gcagttcaac
     1201 agaccgggaa gggctggatt tgctgaggat gaaggtggag gaaggtgatg tcattctggt
     1261 taagaagctc gaccgtcttg gccgcgacac tgccgatatg atccaactga taaaggaatt
```

1321 tgacgctcag ggcgtggcag tccggttcat tgatgacggg atcagtaccg acggtgatat
1381 ggggcaaatg gtggtcacca tcctgtcggc tgtggcacag gctgaacgcc ggaggatcct
1441 agaacgcacg aatgagggcc gacaggaagc aaagctgaaa ggaatcaaat ttggccgcag
1501 gcgtaccgtg gacaggaacg tcgtgctgac gcttcatcag aagggcactg gtgcaacgga
1561 aattgctcat cagctcagta ttgcccgctc cacggtttat aaaattcttg aagacgaaag
1621 ggcctcgtga tacgcctatt tttataggtt aatgtcatga taataatggt ttcttagacg
1681 tcaggtggca cttttcgggg aaatgtgcgc ggaaccccta tttgtttatt tttctaaata
1741 cattcaaata tgtatccgct catgagacaa taaccctggt aaatgcttca ataatattga
1801 aaaaggaaga gtatgagtat tcaacatttt cgtgtcgccc ttattccctt ttttgcggca
1861 ttttgccttc ctgtttttgc tcacccagaa acgctggtga aagtaaaaga tgctgaagat
1921 cagttgggtg cacgagtggg ttacatcgaa ctggatctca acagcggtaa gatccttgag
1981 agttttcgcc ccgaagaacg ttttccaatg atgagcactt ttaaagttct gctatgtggt
2041 gcggtattat cccgtgttga cgccgggcaa gagcaactcg gtcgccgcat acactattct
2101 cagaatgact tggttgagta ctcaccagtc acagaaaagc atcttacgga tggcatgaca
2161 gtaagagaat tatgcagtgc tgccataacc atgagtgata acactgctgc caacttactt
2221 ctgacaacga tcggaggacc gaaggagcta accgcttttt tgcacaacat gggggatcat
2281 gtaactcgcc ttgatcgttg ggaaccggag ctgaatgaag ccataccaaa cgacgagcgt
2341 gacaccacga tgcctgcagc aatggcaaca acgttgcgca aactattaac tggcgaacta
2401 cttactctag cttcccggca acaattaata gactggatgg aggcggataa agttgcagga
2461 ccacttctgc gctcggccct tccggctggc tggtttattg ctgataaatc tggagccggt
2521 gagcgtgggt ctcgcggtat cattgcagca ctggggccag atggtaagcc ctcccgtatc
2581 gtagttatct acacgacggg gagtcaggca actatggatg aacgaaatag acagatcgct
2641 gagataggtg cctcactgat taagcattgg taactgtcag accaagttta ctcatatata
2701 ctttagattg atttaaaact tcatttttaa tttaaaagga tctaggtgaa gatcctttat
2761 gaacatcaac gatgccctca cctccatcct ggcctcaaaa aaataccgcg ccctttgccc
2821 ggataccgtg cggcgcatcc tgactgagga atgggggcgg cataaatccc ccaaacagac
2881 cgtagaggct gcacgcaccc ggctgcatgg aatttgcggg gcatatgtca ccccggaatc
2941 gctcaaggct gctgccgccg cgctttctgc gggcgatgta aaaaaggcat tgtcgctgca
3001 tgcctccacc aaggagcgac tggccgagct ggataccctg tacgatttta tcttttcagc
3061 cgaaactccc cgccgcgtgc tggatatcgc ctgcggtctt aaccccttgg cgctatacga
3121 gcgcggcatt gcatccgtgt ggggctgtga tatccaccag ggattggggg atgtcatcac
3181 ccccttttgct agggaaaaag attgggattt tacctttgcc ctgcaggatg tgctgtgtgc
3241 gccgcccgcc gaagccggcg acctggcgct gatttttaag cttttgcccc tgctggagcg
3301 ggagcaggcc ggttctgcca tggcactttt acaatccctc aataccccgc gcatggctgt
3361 cagctttccc acgcgtagtt taggcgggcg tggaaaaggc atggaggcga actacgccgc
3421 atggttcgag ggcggcttgc ccgccgagtt tgagattgag gataaaaaga ccatcggaac
3481 agaacttata tacttgataa aaaagaatgg ataagccaat cagaaggcaa atcatctaaa
3541 aaagaggaac gaaaaatatt tttcgttcct cttttgttct atcgaacaag cacactacga
3601 ctgctgtttt tgaagcagcc ttttatagct cagctcgatg cccaacgccc ccagcggcgc
3661 tgtaagtata atggcaagaa ccgccaccgc aagtatggtt tccccactgc caagccccat
3721 cgccagcgga atagcgccga ttgcagcctg caccgttgcc ttgggcagat aggcaatcat
3781 gcagaacagg cgctccttcc gggataggtc tgtacccagc gtactcatcc acacgcccag
3841 catgcgaaac agcagcgcag ctgtgatcag cagcatgccg cttagcccgg cggagaaaag
3901 gtaacggatg ttgaccgttg cacccaccag cacaaacagc cagatttctg ccgccaccca
3961 aacctttgaa aatttgccgg atatacgctt tgcaaccggg atgaaaaccg ccactgcgcc
4021 gttaccaccg ctgcgttcgg tcaaggttct ggaccagttg cgtgagcgca tacgctactt
4081 gcattacagc ttaccaaccg aacaggctta tgtccactgg gttcgtgcct tcatccgttt
4141 ccacggtgtg cgtcacccgg caaccttggg cagcagcgaa gtcgaggcat ttctgtcctg
4201 gctggcgaac gagcgcaagg tttcggtctc cacgcatcgt caggcattgg cggccttgct
4261 gttcttctac ggcaaggtgc tgtgcacgga tctgccctgg cttaaggaga tcggaagacc
4321 tcgcccgtcg cggcgcttgc cggtggtgct gaccccggat gaagtggttc gcatcctcgg
4381 ttttctggaa ggcgagcatc gtttgttcgc ccagcttctg tatggaacgg gcatgcggat
4441 cagtgagggt ttgcaactgc gggtcaagga tctggatttc gatcacggca cgatcatcgt
4501 gcgggagggc aagggctcca aggatcgggc cttgatgtta cccgagagct tggcacccag
4561 cctgcgcgag cagctgtcgc gtgcacgggc atggtggctg aaggaccagg ccgagggccg
4621 cagcggcgtt gcgcttcccg acgcccttga gcggaagtat ccgcgcgccg ggcattcctg
4681 gcggcactgt tgcaaatagt cggtggtgat aaacttatca gccgtccccg caccggagcg
4741 ctgcgtgccg cgcctcgcca catcccggcg gcaagccgcg ggatgcgcgc cagtgccgtc
4801 cgcccacacc ggttcgcggt acgcgcgcca cgcgcccgag cgcacgctgc tgtacgcgtt
4861 ggtagaggcg cactacccgg acttcattgc acggatcgaa gcggagggcc gctcgctgcc
4921 cgggtatgtc cgcgaggcgt tcgatgccta cctgcgttgc ggcgtactcg agcacggctt

4981 cctgcgggtg gtgtgcgagc actgccgtgc agagaggctg gtggccttct cctgcaagaa
5041 gcgcgggttc tgcccgagtt gcggcgcgcg acgcatggcc gagagtgcgc ggcacctggt
5101 cgaggaggtg ttcggcccgc ggcctgtgcg gcaatgggtg ctgagctttc cgtacccctt
5161 gcgtttcctg ttcgccagca agccagaagc cattggcccg gtgctgggca tcgtgcagcg
5221 cgtgatcgcc ggctggttgg ccgatcaagc cggcatcgac cgcgccagcg cccagtgcgg
5281 cgcggtgacg ctgatccagc gtttcggcag cgcgctgaac ctgaacatcc acttccacat
5341 gctgtggctc gacggcgtgt acgtggaagc caccgagctg ccgcggcgcg aactgcgcct
5401 gcaccgcgcc cgtgcgccca ccaccgcgca gttgacccag ctggcagcta ccatcgcgca
5461 ccgggtgtgt cggcacctga cgcgcaaagg ctggctcgaa ggggagggcg aatcggcctt
5521 cctggcagac agcgctgcag gcgacgacag catggatggg ctgcggatga gttcgatcac
5581 ctaccgcatc gccaccggcc gcgacgctgg ctgcaaggtc gtcacgctgc aaacgctgcc
5641 cggtgacgcc ggttcgctgg agggcgaagc cggcaaggtc ggcggcttct cactgcatgc
5701 cggcgtggcg gccgaagcac acgaaagcca caagcgggaa aagctgtgcc gctacatcac
5761 gcgcccggcg atcagcgaga agcggctgtc gatagcgctc cagggcaggg tgcgttacca
5821 gctcaagacc ccgtggcgca atggcaccac gcatgtggaa tgggatccgg tggatttcat
5881 cgccaagctg gcggcgctgg tcccgccacc tcgcgcgcat ctcacccgct tccacggcgt
5941 attcgccccg aatgcaaacc tgcgtgcgca gctgacgccc tcggggcgcg gcaagcggcc
6001 tgcgggcgat gcggcgccag tggacgtcag cgcccacgac gcgccgcgca gccccgagga
6061 gaagcgccgt gcgatgagct gggcgcaacg gctcaagcgg gtcttttcca tcgacgtcac
6121 cgcctgcgtc cactgcggtg gcaccgtgcg gatcgtcgcc agcatcgagg aacccaccgc
6181 catccgcgcc atcctcgccc acttcgagaa gcacggcgcg cgggaagaag cgcactacag
6241 gcccgcagcg cgcgcgccgc cagtgcaagc cgcgtgacga tctgccggct gcacagccga
6301 cggcgaaacc ggaatccgag ccgatgcggc cacgatccgc agggcggcgc tcggcccgct
6361 gtcgggaatc agcgaagcat ggctgctgac aacgccgctg cgtggccccg cgatgccgaa
6421 atcccactca cagacgtccg atccgtgccc aaaacggggc ttgcgcgacc gccgcctacc
6481 cagcagactg cccgaaaagg gcgtttgaac ttcctatacg caacccctcg cgcatgtcgc
6541 accgccgcct gaaagagacc atcggcgtgc gctacaacga tttcggcgtg gtcgagccca
6601 tcgtcgccga catccgcgcc atgctggcca cgcacgacgg catcgacacc acgcagacgc
6661 tgatcgtgaa cttcaacgcg ttcgggccga gctcgctgga catcatggtc tacaccttca
6721 ccaagaccac ggtgtgggtc accttccacg agatcaagca ggatgtgctg ctgcgcatcg
6781 ggcgcatcgt cgagtcgcat ggcgccgaga tcgcgtttcc gacgcagacc gtctacctcg
6841 cgcagccaga agagccgcct gaggccatgc gacctgcagc cgcccccgcca gcagcgcgct
6901 gaatccagcg cggtccggac gcgagcgggt caaccagatg cgagcgctgg gcgcttgcgc
6961 agcaggcgcg ccaccagccc agcggccacc agcaccagca ccgcgccggc ccaggccgtg
7021 gcctgcagcg catcggtgaa gcccgcgcgt gcggccgcca gcaaggccgc gccctgccac
7081 gccggcaggg tgtcggccag gtgcacggcg cccccgagcg aggcaccggc cgtctgcagc
7141 gcatcggccg gcaggccggg cagcgccgcg ctggtcagcg cctgccggta gaccaccagg
7201 ccgacgctgc cgaacagcgc gatgcccagc gcgccgctga attcggacac cgtctccgac
7261 aaggccgagg ccgcgcccgc gcgctcggac ggcgcgctgg tgatgatgat ctcgttgccg
7321 atggtgaaca ccggcgccag gcccaggccc ttgacgatcg tggccggcac cagccaccac
7381 aggccctgcc ccagccccag cacggcgaag ccgaacgccg ctgccgacag gcccacgacg
7441 aggatgcgcg ccgccggcca gcgcgccgcg agctgcggcg acaacagcga accgatgacg
7501 aagcacaggg agcagggcag cgtggccagc ccggcctgca gcggcgacag ccccagcacg
7561 agctgcaggt actgcgtcat gaagatgtag acgccgaaca tggccagcgc ggccagcgca
7621 tacgccgcca gcgccgcgcg gaacagcgcg tgcgcgaaca gccgcaggtc cagcagcggg
7681 taggcgatgt ggccctggcg gcgcaggaac agcgccccga ccgccagccc ggccagcagc
7741 gcagccatcg aggcgaggcc ctctccatgc tcggccaact gcttgagccc gtagatcgtc
7801 agcagcaccg ccgccagcga cagcagcacg ctggccaggt ccaggtgccc cgcgtccgga
7861 tcacgatact cgggcaggaa gcgagggccg agcgccagcg tcagcagcat caccggcacg
7921 ttgagccaga acacggcgcc ccagtggaag aactccagca acacgccgcc gaccagcgga
7981 ccgatcgcgc tgcccagcga aaacgcggcg atccacacgc cgatggcgaa ctggcgctgg
8041 cgcgggtcgt ggaacatgtt gcggaccagc gccatggtgg acggcgcgat ggtggcgccg
8101 gccaggccga gcaaggcgcg cgcccgcgatc aacagcgcgg cggtatcggc cagcgccgcg
8161 agcaccgagg cgaatgcgaa gaacgccgcg ccgatcaaca acagccggcg ccggccgatg
8221 cggtcgccca gcgtgcccat ggtgatcagg aagccggcga cgaagaagcc gtagatgtcc
8281 aggatccaga gaagctgggc gctggagggc tgcagttcac ggctgagcac cggcagcgcc
8341 aggttcagca ccgtgaggtc catggcgtag accaggcacg gcagggcgac cacggccagg
8401 ccgatccatt cgcggcgggt ggccttccga cgatccgctg cggtgtcgtg gagcgtggcg
8461 gacatcaacg cgcgcatttc gacatgccgt cgatggcaac acccatccgc attcaggcgc
8521 gcgtgacacg catgcctgtt gcccgtcgca catcaggggg cacggtcgta gccggccagc
8581 agctgaagta ctgacctcat tttttccttc attcatttca aacctctttt taatgtttta

```
     8641 ggttatcgcc agctcggcaa cttgatactc tggtgaagcc caactttgtt ttagggcgac
     8701 tgccctgctg cgtaacatcg ttgctgctcc ataacatcaa acatcgaccc acggcgtaac
     8761 gcgcttgctg cttggatgcc cgaggcatag actgtacaaa aaaacagtca taacaagcca
     8821 tgaaaaccgc cactgcgccg ttaccaccgc tgcgttcatg aaaaccgcca ctgcgccgtt
     8881 accaccgctg cgttcggtca aggttctgga ccagttgcgt gagcgcatac gctacttgca
     8941 ttacagctta ccaaccgaac aggcttatgt ccactgggtt cgtgccttca tccgtttcca
     9001 cggtgtgcgt cacccggcaa ccttgggcag cagcgaagtc gaggcatttc tgtcctggct
     9061 ggcgaacgag cgcaaggttt cggtctccac gcatcgtcag gcattggcgg ccttgctgtt
     9121 cttctacggc aaggtgctgt gcacggatct gccctggctt aaggagatcg gaagacctcg
     9181 cccgtcgcgg cgcttgccgg tggtgctgac cccggatgaa gtggttcgca tcctcggttt
     9241 tctggaaggc gagcatcgtt tgttcgccca gcttctgtat ggaacgggca tgcggatcag
     9301 tgagggtttg caactgcggg tcaaggatct ggatttcgat cacggcacga tcatcgtgcg
     9361 ggagggcaag ggctccaagg atcgggcctt gatgttaccc gagagcttgg cacccagcct
     9421 gcgcgagcag ctgtcgcgtg cacgggcatg gtggctgaag gaccaggccg agggccgcag
     9481 cggcgttgcg cttcccgacg cccttgagcg gaagtatccg cgcgccgggc attcctggcg
     9541 gcactgttgc aaatagtcgg tggtgataaa cttatcatcc ccttttgctg atggagctgc
     9601 acatgaaccc attcaaaggc cggcattttc agcgtgacat cattctgtgg gccgtacgct
     9661 ggtactgcaa atacggcatc agttaccgtg agctgcagga gatgctggct gaacgcggag
     9721 tgaatgtcga tcactccacg atttaccgct gggttcagcg ttatgcgcct gaaatggaaa
     9781 aacggctgcg ctggtactgg cgtaaccctt ccgatctttg cccgtggcac atggatgaaa
     9841 cctacgtgaa ggtcaatggc cgctgggcgt atctgtaccg ggccgtcgac agccggggcc
     9901 gcactgtcga tttttatctc tcctcccgtc gtaacagcaa agctgcatac cggtttctgg
     9961 gtaaaatcct caacaacgtg aagaagtggc agatcccgcg attcatcaac acggataaag
    10021 cgcccgccta tggtcgcgcg cttgctctgc tcaaacgcga aggccggtgc ccgtctgacg
    10081 ttgaacaccg acagattaag taccggaaca acgtgattga atgcgatcat ggcaaactga
    10141 aacggataat cggcgccacg ctgggattta aatccatgaa gacggcttac gccaccatca
    10201 aaggtattga ggtgatgcgt gcactacgca aaggccaggc ctcagcattt tattatggtg
    10261 atcccctggg cgaaatgcgc ctggtaagca gagtttttga aatgtaaggc ctttgaataa
    10321 gacaaaaggc tgcctcatcg ctaactttgc aacagtgcc
//

LOCUS       KR259133                4656 bp    DNA     circular BCT 08-JUL-2015
DEFINITION  Klebsiella pneumoniae strain KP3764 plasmid PKP3764, complete
            sequence.
ACCESSION   KR259133
VERSION     KR259133
KEYWORDS    .
SOURCE      Klebsiella pneumoniae
  ORGANISM  Klebsiella pneumoniae
            Bacteria; Proteobacteria; Gammaproteobacteria; Enterobacteriales;
            Enterobacteriaceae; Klebsiella.
REFERENCE   1  (bases 1 to 4656)
  AUTHORS   Wang,D.
  TITLE     A plasmid from isolate KP3764 of Klebsiella pneumoniae involving
            novel qepA3 genes from patient's blood in Taizhou municipal
            hospital of China
  JOURNAL   Unpublished
REFERENCE   2  (bases 1 to 4656)
  AUTHORS   Wang,D.
  TITLE     Direct Submission
  JOURNAL   Submitted (22-APR-2015) Department of Clinical Lab Medicine,
            Taizhou Municipal Hospital and Institute of Molecular Diagnostics
            of Taizhou University, 381 East Rd. of Zhongshan in Jiaojiang of
            Taizhou, Taizhou, Zhejiang 318000, China
COMMENT     ##Assembly-Data-START##
            Sequencing Technology :: Sanger dideoxy sequencing
            ##Assembly-Data-END##
FEATURES             Location/Qualifiers
     source          1..4656
                     /organism="Klebsiella pneumoniae"
                     /mol_type="genomic DNA"
```

```
                     /strain="KP3764"
                     /host="Homo sapiens"
                     /db_xref="taxon:573"
                     /plasmid="PKP3764"
                     /country="China: Taizhou municipal hospital of Zhejiang
                     province"
                     /lat_lon="28 N 122 E"
                     /collection_date="15-Nov-2013"
                     /collected_by="Dongguo Wang"
                     /note="genotype: tnpA;qepA3;dfr2;intI1-groEL;tnpA"
     gene            52..768
                     /gene="tnpA"
                     /note="insertion sequence:IS26; tnpA gene; transposase
                     IS26"
                     /pseudo
     gene            complement(1225..2760)
                     /gene="qepA3"
     CDS             complement(1225..2760)
                     /gene="qepA3"
                     /note="quinolone efflux pump"
                     /codon_start=1
                     /transl_table=11
                     /product="QepA3"
                     /protein_id="AKP49185"
                     /translation="MSATLHDTAADRRKATRREWIGLAVVALPCLVYAMDLTVLNLAL
                     PVLSRELQPSSAQLLWILDIYGFFVAGFLITMGTLGDRIGRRRLLLIGAAFFAFASVL
                     AALADTAALLIAARALLGLAGATIAPSTMALVRNMFHDPRQRQFAIGVWIAAFSLGSA
                     IGPLVGGVLLEFFHWGAVFWLNVPVMLLTLALGPRFLPEYRDPDAGHLDLASVLLSLA
                     AVLLTIYGLKQLAEHGEGLASMAALLAGLAVGALFLRRQGHIAYPLLDLRLFAHALFR
                     AALAAYALAALAMFGVYIFMTQYLQLVLGLSPLQAGLATLPCSLCFVIGSLLSPQLAA
                     RWPAARILVVGLSAAAFGFAVLGLGQGLWWLVPATIVKGLGLAPVFTIGNEIIITSAP
                     SERAGAASALSETVSEFSGALGIALFGSVGLVVYRQALTSAALPGLPADALQTAGASL
                     GGAVHLADTLPAWQGAALLAAARAGFTDALQATAWAGAVLVLVAAGLVARLLRKRPAL
                     ASG"
     gene            2819..2929
                     /gene="dfr2"
                     /note="trimethoprim dihydrofolate reductase; dfr2 gene;
                     Dfr2"
                     /pseudo
     gene            3155..3853
                     /gene="integrase fusion groEL/intI1"
                     /note="similar to chaperonin/integrase fusion protein;
                     integrase fusion gene groEL/intI1; integrase fusion
                     protein"
                     /pseudo
     gene            3888..4604
                     /gene="tnpA"
                     /note="tnpA gene; transposase IS26"
                     /pseudo
ORIGIN
        1 ggcactgttg caaatagtcg gtggtgataa acttatcatc cccttttgct gatggagctg
       61 cacatgaacc cattcaaagg ccggcatttt cagcgtgaca tcattctgtg ggccgtacgc
      121 tggtactgca aatacggcat cagttaccgt gagctgcagg agatgctggc tgaacgcgga
      181 gtgaatgtcg atcactccac gatttaccgc tgggttcagc gttatgcgcc tgaaatggaa
      241 aaacggctgc gctggtactg gcgtaaccct tccgatcttt gcccgtggca catggatgaa
      301 acctacgtga aggtcaatgg ccgctgggcg tatctgtacc gggccgtcga cagccggggc
      361 cgcactgtcg atttttatct ctcctcccgt cgtaacagca aagctgcata ccggtttctg
      421 ggtaaaatcc tcaacaacgt gaagaagtgg cagatcccgc gattcatcaa cacggataaa
      481 gcgcccgcct atggtcgcgc gcttgctctg ctcaaacgcg aaggccggtg cccgtctgac
      541 gttgaacacc gacagattaa gtaccggaac aacgtgattg aatgcgatca tggcaaactg
      601 aaacggataa tcggcgccac gctgggattt aaatccatga agacggctta cgccaccatc
```

661 aaaggtattg aggtgatgcg tgcactacgc aaaggccagg cctcagcatt ttattatggt
721 gatcccctgg gcgaaatgcg cctggtaagc agagtttttg aaatgtaagg cctttgaata
781 gactgcccga aaagggcgtt tgaacttcct atacgcaacc cctcgcgcat gtcgcaccgc
841 cgcctgaaag agaccatcgg cgtgcgctac aacgatttcg gcgtggtcga gcccatcgtc
901 gccgacatcc gcgccatgct ggccacgcac gacggcatcg acaccacgca gacgctgatc
961 gtgaacttca acgcgttcgg gccgagctcg ctggacatca tggtctacac cttcaccaag
1021 accacggtgt gggtcacctt ccacgagatc aagcaggatg tgctgctgcg catcgggcgc
1081 atcgtcgagt cgcatggcgc cgagatcgcg tttccgacgc agaccgtcta cctcgcgcag
1141 ccagaagagc cgcctgaggc catgcgacct gcagccgccc cgccagcagc gcgctgaatc
1201 cagcgcggtc cggacgcgag cgggtcaacc agatgcgagc gctgggcgct tgcgcagcag
1261 gcgcgccacc agcccagcgg ccaccagcac cagcaccgcg ccggcccagg ccgtggcctg
1321 cagcgcatcg gtgaagcccg cgcgtgcggc cgccagcaag gccgcgccct gccacgccgg
1381 cagggtgtcg gccaggtgca cggcgccccc gagcgaggca ccggccgtct gcagcgcatc
1441 ggccggcagg ccgggcagcg ccgcgctggt cagcgcctgc cggtagacca ccaggccgac
1501 gctgccgaac agcgcgatgc ccagcgcgcc gctgaattcg gacaccgtct ccgacaaggc
1561 cgaggccgcg cccgcgcgct cggacggcgc gctggtgatg atgatctcgt tgccgatggt
1621 gaacaccggc gccaggccca ggcccttgac gatcgtggcc ggcaccagcc accacaggcc
1681 ctgccccagc cccagcacgg cgaagccgaa cgccgctgcc gacaggccca cgacgaggat
1741 gcgcgccgcc ggccagcgcg ccgcgagctg cggcgacaac agcgaaccga tgacgaagca
1801 cagggagcag ggcagcgtgg ccagcccggc ctgcagcggc gacagcccca gcacgagctg
1861 caggtactgc gtcatgaaga tgtagacgcc gaacatggcc agcgcggcca gcgcatacgc
1921 cgccagcgcc gcgcggaaca gcgcgtgcgc gaacagccgc aggtccagca gcgggtaggc
1981 gatgtggccc tggcggcgca ggaacagcgc cccgaccgcc agcccggcca gcagcgcagc
2041 catcgaggcg aggccctctc catgctcggc caactgcttg agcccgtaga tcgtcagcag
2101 caccgccgcc agcgacagca gcacgctggc caggtccagg tgccccgcgt ccggatcacg
2161 atactcgggc aggaagcgag ggccgagcgc cagcgtcagc agcatcaccg gcacgttgag
2221 ccagaacacg gcgccccagt ggaagaactc cagcaacacg ccgccgacca gcggaccgat
2281 cgcgctgccc agcgaaaacg cggcgatcca cacgccgatg gcgaactggc gctggcgcgg
2341 gtcgtggaac atgttgcgga ccagcgccat ggtggacggc gcgatggtgg cgccggccag
2401 gccgagcaag gcgcgcgccg cgatcaacag cgcggcggta tcggccagcg ccgcgagcac
2461 cgaggcgaat gcgaagaacg ccgcgccgat caacaacagc cggcgccggc cgatgcggtc
2521 gcccagcgtg cccatggtga tcaggaagcc ggcgacgaag aagccgtaga tgtccaggat
2581 ccagagaagc tgggcgctgg agggctgcag ttcacggctg agcaccggca gcgccaggtt
2641 cagcaccgtg aggtccatgg cgtagaccag gcacggcagg gcgaccacgg ccaggccgat
2701 ccattcgcgg cgggtggcct tccgacgatc cgctgcggtg tcgtggagcg tggcggacat
2761 actcaaaact tccgggacaa gaatgcttta acgacgaagg caacgcgcgc atttcgacat
2821 gccgtcgatg gcaacaccca tccgcattca ggcgcgcgtg acacgcatgc ctgttgcccg
2881 tcgcacatca gggggcacgg tcgtagccgg ccagcagctg aagtactgac ctcatttttt
2941 ccttcattca tttcaaacct ctttttaatg ttttaggtta tcgccagctc ggcaacttga
3001 tactctggtg aagcccaact ttgttttagg gcgactgccc tgctgcgtaa catcgttgct
3061 gctccataac atcaaacatc gacccacggc gtaacgcgct tgctgcttgg atgcccgagg
3121 catagactgt acaaaaaaac agtcataaca agccatgaaa accgccactg cgccgttacc
3181 accgctgcgt tcggtcaagg ttctggacca gttgcgtgag cgcatacgct acttgcatta
3241 cagcttacca accgaacagg cttatgtcca ctgggttcgt gccttcatcc gtttccacgg
3301 tgtgcgtcac ccggcaacct tgggcagcag cgaagtcgag gcatttctgt cctggctggc
3361 gaacgagcgc aaggtttcgg tctccacgca tcgtcaggca ttggcggcct tgctgttctt
3421 ctacggcaag gttctgtgca cggatctgcc ctggcttcag gagatcggaa gacctcggcc
3481 gtcgcggcgc ttgccggtgg tgctgacccc ggatgaagtg gttcgcatcc tcggttttct
3541 ggaaggcgag catcgtttgt tcgcccagct tctgtatgga acgggcatgc ggatcagtga
3601 gggtttgcaa ctgcgggtca aggatctgga tttcgatcac ggcacgatca tcgtgcggga
3661 gggcaagggc tccaaggatc gggccttgat gttacccgag agcttggcac ccagcctgcg
3721 cgagcagctg tcgcgtgcac gggcatggtg gctgaaggac caggccgagg gccgcagcgg
3781 cgttgcgctt cccgacgccc ttgagcggaa gtatccgcgc gccgggcatt cctggcggca
3841 ctgttgcaaa tagtcggtgg tgataaactt atcatcccct tttgctgatg gagctgcaca
3901 tgaacccatt caaaggccgg cattttcagc gtgacatcat tctgtgggcc gtacgctggt
3961 actgcaaata cggcatcagt taccgtgagc tgcaggagat gctggctgaa cgcggagtga
4021 atgtcgatca ctccacgatt taccgctggg ttcagcgtta tgcgcctgaa atggaaaaac
4081 ggctgcgctg gtactggcgt aacccttccg atctttgccc gtggcacatg gatgaaacct
4141 acgtgaaggt caatggccgc tgggcgtatc tgtaccgggc cgtcgacagc cggggccgca
4201 ctgtcgattt ttatctctcc tcccgtcgta acagcaaagc tgcataccgg tttctgggta
4261 aaatcctcaa caacgtgaag aagtggcaga tcccgcgatt catcaacacg gataaagcgc

```
     4321 ccgcctatgg tcgcgcgctt gctctgctca aacgcgaagg ccggtgcccg tctgacgttg
     4381 aacaccgaca gattaagtac cggaacaacg tgattgaatg cgatcatggc aaactgaaac
     4441 ggataatcgg cgccacgctg ggatttaaat ccatgaagac ggcttacgcc accatcaaag
     4501 gtattgaggt gatgcgtgca ctacgcaaag gccaggcctc agcattttat tatggtgatc
     4561 ccctgggcga aatgcgcctg gtaagcagag tttttgaaat gtaaggcctt tgaataagac
     4621 aaaaggctgc ctcatcgcta actttgcaac agtgcc
//

LOCUS       KR259134                6848 bp    DNA     circular BCT 08-JUL-2015
DEFINITION  Enterobacter cloacae strain ECL3786 plasmid pECL3786, complete
            sequence.
ACCESSION   KR259134
VERSION     KR259134
KEYWORDS    .
SOURCE      Enterobacter cloacae
  ORGANISM  Enterobacter cloacae
            Bacteria; Proteobacteria; Gammaproteobacteria; Enterobacteriales;
            Enterobacteriaceae; Enterobacter; Enterobacter cloacae complex.
REFERENCE   1  (bases 1 to 6848)
  AUTHORS   Wang,D.
  TITLE     A plasmid from isolate ECL3786 of Enterobacter Cloacae involving
            16S rRNA methylases and novel qepA3 genes from patient's wound
            secretion of chest in Taizhou municipal hospital of China
  JOURNAL   Unpublished
REFERENCE   2  (bases 1 to 6848)
  AUTHORS   Wang,D.
  TITLE     Direct Submission
  JOURNAL   Submitted (23-APR-2015) Department of Clinical Lab Medicine,
            Taizhou Municipal Hospital and Institute of Molecular Diagnostics
            of Taizhou University, 381 East Rd. of Zhongshan in Jiaojiang of
            Taizhou, Taizhou, Zhejiang 318000, China
COMMENT     ##Assembly-Data-START##
            Sequencing Technology :: Sanger dideoxy sequencing
            ##Assembly-Data-END##
FEATURES             Location/Qualifiers
     source          1..6848
                     /organism="Enterobacter cloacae"
                     /mol_type="genomic DNA"
                     /strain="ECL3786"
                     /host="Homo sapiens"
                     /db_xref="taxon:550"
                     /plasmid="pECL3786"
                     /country="China: Taizhou municipal hospital of Zhejiang
                     province"
                     /lat_lon="28 N 122 E"
                     /collection_date="16-Feb-2014"
                     /collected_by="Dongguo Wang"
                     /note="genotype: tnpA;rmtB;qepA3;dfr2;intI1-groEL;tnpA"
     gene            42..758
                     /gene="tnpA"
                     /note="insertion sequence:IS26; tnpA gene; transposase
                     IS26"
                     /pseudo
     gene            980..1735
                     /gene="rmtB"
     CDS             980..1735
                     /gene="rmtB"
                     /codon_start=1
                     /transl_table=11
                     /product="16S rRNA methylase"
                     /protein_id="AKP49186"
```

```
                     /translation="MNINDALTSILASKKYRALCPDTVRRILTEEWGRHKSPKQTVEA
                     ARTRLHGICGAYVTPESLKAAAAALSAGDVKKALSLHASTKERLAELDTLYDFIFSAE
                     TPRRVLDIACGLNPLALYERGIASVWGCDIHQGLGDVITPFAREKDWDFTFALQDVLC
                     APPAEAGDLALIFKLLPLLEREQAGSAMALLQSLNTPRMAVSFPTRSLGGRGKGMEAN
                     YAAWFEGGLPAEFEIEDKKTIGTELIYLIKKNG"
     gene            complement(3417..4952)
                     /gene="qepA3"
     CDS             complement(3417..4952)
                     /gene="qepA3"
                     /note="quinolone efflux pump"
                     /codon_start=1
                     /transl_table=11
                     /product="QepA3"
                     /protein_id="AKP49187"
                     /translation="MSATLHDTAADRRKATRREWIGLAVVALPCLVYAMDLTVLNLAL
                     PVLSRELQPSSAQLLWILDIYGFFVAGFLITMGTLGDRIGRRRLLLIGAAFFAFASVL
                     AALADTAALLIAARALLGLAGATIAPSTMALVRNMFHDPRQRQFAIGVWIAAFSLGSA
                     IGPLVGGVLLEFFHWGAVFWLNVPVMLLTLALGPRFLPEYRDPDAGHLDLASVLLSLA
                     AVLLTIYGLKQLAEHGEGLASMAALLAGLAVGALFLRRQGHIAYPLLDLRLFAHALFR
                     AALAAYALAALAMFGVYIFMTQYLQLVLGLSPLQAGLATLPCSLCFVIGSLLSPQLAA
                     RWPAARILVVGLSAAAFGFAVLGLGQGLWWLVPATIVKGLGLAPVFTIGNEIIITSAP
                     SERAGAASALSETVSEFSGALGIALFGSVGLVVYRQALTSAALPGLPADALQTAGASL
                     GGAVHLADTLPAWQGAALLAAARAGFTDALQATAWAGAVLVLVAAGLVARLLRKRPAL
                     ASG"
     gene            5011..5121
                     /gene="dfr2"
                     /note="trimethoprim dihydrofolate reductas; dfr2 gene;
                     Dfr2"
                     /pseudo
     gene            5347..6045
                     /gene="integrase fusion groEL/intI1"
                     /note="similar to chaperonin/integrase fusion protein;
                     integrase fusion gene groEL/intI1; integrase fusion
                     protein"
                     /pseudo
     gene            6080..6796
                     /gene="tnpA"
                     /note="tnpA gene; transposase IS26"
                     /pseudo
ORIGIN
        1 caaatagtcg gtggtgataa acttatcatc cccttttgct gatggagctg cacatgaacc
       61 cattcaaagg ccggcatttt cagcgtgaca tcattctgtg ggccgtacgc tggtactgca
      121 aatacggcat cagttaccgt gagctgcagg agatgctggc tgaacgcgga gtgaatgtcg
      181 atcactccac gatttaccgc tgggttcagc gttatgcgcc tgaaatggaa aaacggctgc
      241 gctggtactg gcgtaaccct tccgatcttt gcccgtggca catggatgaa acctacgtga
      301 aggtcaatgg ccgctgggcg tatctgtacc gggccgtcga cagccggggc cgcactgtcg
      361 atttttatct ctcctcccgt cgtaacagca aagctgcata ccggtttctg ggtaaaatcc
      421 tcaacaacgt gaagaagtgg cagatcccgc gattcatcaa cacggataaa gcgcccgcct
      481 atggtcgcgc gcttgctctg ctcaaacgcg aaggccggtg cccgtctgac gttgaacacc
      541 gacagattaa gtaccggaac aacgtgattg aatgcgatca tggcaaactg aaacggataa
      601 tcggcgccac gctgggattt aaatccatga agacggctta cgccaccatc aaaggtattg
      661 aggtgatgcg tgcactacgc aaaggccagg cctcagcatt ttattatggt gatcccctgg
      721 gcgaaatgcg cctggtaagc agagtttttg aaatgtaagg cctttgaata agacaaaagg
      781 ctgcctcatc gctaactttg caacagtgcc ctgtcagacc aagtttactc atatatactt
      841 tagattgatt taaaacttca tttttaattt aaaaggatct aggtgaagat cctttttgat
      901 aatctcatga ccaaaatccc ttaacgtgag ttttcgttcc actgagcgtc agaccccgaa
      961 aacgatttag gagacaccga tgaacatcaa cgatgccctc acctccatcc tggcctcaaa
     1021 aaaataccgc gccctttgcc cggataccgt gcggcgcatc ctgactgagg aatgggggcg
     1081 gcataaatcc cccaaacaga ccgtagaggc tgcacgcacc cggctgcatg gaatttgcgg
     1141 ggcatatgtc accccggaat cgctcaaggc tgctgccgcc gcgctttctg cgggcgatgt
     1201 aaaaaaggca ttgtcgctgc atgcctccac caaggagcga ctggccgagc tggataccct
```

1261 gtacgatttt atcttttcag ccgaaactcc ccgccgcgtg ctggatatcg cctgcggtct
1321 taacccccttg gcgctatacg agcgcggcat tgcatccgtg tggggctgtg atatccacca
1381 gggattgggg gatgtcatca cccccctttgc tagggaaaaa gattgggatt ttaccttttgc
1441 cctgcaggat gtgctgtgtg cgccgcccgc cgaagccggc gacctggcgc tgatttttaa
1501 gcttttgccc ctgctggagc gggagcaggc cggttctgcc atggcacttt tacaatccct
1561 caatacccccg cgcatggctg tcagctttcc cacgcgtagt ttaggcgggc gtggaaaagg
1621 catggaggcg aactacgccg catggttcga gggcggcttg cccgccgagt ttgagattga
1681 ggataaaaag accatcggaa cagaacttat atacttgata aaaaagaatg gataagccaa
1741 tcagaaggca aatcatctaa aaaagaggaa cgaaaaatat ttttcgttcc tcttttgttc
1801 tatcgaacaa gcacactacg actgctgttt ttgaagcagc cttttatagc tcagctcgat
1861 gcccaacgcc cccagcggcg ctgtaagtat aatggcaaga accgccaccg caagtatggt
1921 ttccccactg ccaagcccca tcgccagcgg aatagcgccg attgcagcct gcaccgttgc
1981 cttgggcaga taggcaatca tgcagaacag gcgctccttc cgggataggt ctgtacccag
2041 cgtactcatc cacacgccca gcatgcgaaa cagcagcgca gctgtgatca gcagcatgcc
2101 gcttagcccg gcggagaaaa ggtaacggat gttgaccgtt gcacccacca gcacaaacag
2161 ccagatttct gccgccaccc aaagctttga aaatttgccg gatatacgct ttgcaaccgg
2221 ggcgttggtt tttaaaaggg tgacgcccat ccccatcacc gccagcaatc cggaaaatag
2281 cgcccctttcg cccagggttt tctccaacgc aaccagcgca aaggatgcgc ccagcagcag
2341 gagcaccttg atcgtatcac gcatatgtac ccgcttaaaa agggctgccg gccggcaagg
2401 tgctgtgcac ggatctgccc tggcagggca tcaacgaaga ccagaacctg ggcatcgcca
2461 tcacccgccg tgcgctggaa gccccgctgc gcgccatcgt ggccaacgcc ggtgaagaac
2521 cgagcgtgat cgtggccaac gtcaaggccg gcgaaggcag ctacggctac aacgccgcca
2581 ccggcgagtt cggcgacatg atcgccatgg gcatcctgga cccgaccaag gtgacccgct
2641 cggccctgca gcacgccgct tccgtcgccg gccttgcgat cacgaccgaa gtggtcgtgg
2701 ccgaagtgcc gaagaaggaa gagccggcca tgccgggtgc tggcggtatg ggcggcatgg
2761 gcggcgatct gccggctgca cagccgacgg cgaaaccgga atccgagccg atgcggccac
2821 gatccgcagg gcggcgctcg gcccgctgtc gggaatcagc gaagcatggc tgctgacaac
2881 gccgctgcgt ggccccgcga tgccgaaatc ccactcacag acgtccgatc cgtgcccaaa
2941 acggggcttg cgcgaccgcc gcctacccag cagactgccc gaaaagggcg tttgaacttc
3001 ctatacgcaa cccctcgcgc atgtcgcacc gccgcctgaa agagaccatc ggcgtgcgct
3061 acaacgattt cggcgtggtc gagcccatcg tcgccgacat ccgcgccatg ctggccacgc
3121 acgacggcat cgacaccacg cagacgctga tcgtgaactt caacgcgttc gggccgagct
3181 cgctggacat catggtctac accttcacca agaccacggt gtgggtcacc ttccacgaga
3241 tcaagcagga tgtgctgctg cgcatcgggc gcatcgtcga gtcgcatggc gccgagatcg
3301 cgtttccgac gcagaccgtc tacctcgcgc agccagaaga gccgcctgag gccatgcgac
3361 ctgcagccgc cccgccagca gcgcgctgaa tccagcgcgg tccggacgcg agcgggtcaa
3421 ccagatgcga gcgctgggcg cttgcgcagc aggcgcgcca ccagcccagc ggccaccagc
3481 accagcaccg cgccggccca ggccgtggcc tgcagcgcat cggtgaagcc cgcgcgtgcg
3541 gccgccagca aggccgcgcc ctgccacgcc ggcagggtgt cggccaggtg cacggcgccc
3601 ccgagcgagg caccggccgt ctgcagcgca tcggccggca ggccgggcag cgccgcgctg
3661 gtcagcgcct gccggtagac caccaggccg acgctgccga acagcgcgat gcccagcgcg
3721 ccgctgaatt cggacaccgt ctccgacaag gccgaggccg cgcccgcgcg ctcggacggc
3781 gcgctggtga tgatgatctc gttgccgatg gtgaacaccg gcgccaggcc caggcccttg
3841 acgatcgtgg ccggcaccag ccaccacagg ccctgcccca gccccagcac ggcgaagccg
3901 aacgccgctg ccgacaggcc cacgacgagg atgcgcgccg ccggccagcg cgccgcgagc
3961 tgcggcgaca acagcgaacc gatgacgaag cacagggagc agggcagcgt ggccagcccg
4021 gcctgcagcg gcgacagccc cagcacgagc tgcaggtact gcgtcatgaa gatgtagacg
4081 ccgaacatgg ccagcgcggc cagcgcatac gccgccagcg ccgcgcggaa cagcgcgtgc
4141 gcgaacagcc gcaggtccag cagcgggtag gcgatgtggc cctggcggcg caggaacagc
4201 gccccgaccg ccagcccggc cagcagcgca gccatcgagg cgaggccctc tccatgctcg
4261 gccaactgct tgagcccgta gatcgtcagc agcaccgccg ccagcgacag cagcacgctg
4321 gccaggtcca ggtgccccgc gtccggatca cgatactcgg gcaggaagcg agggccgagc
4381 gccagcgtca gcagcatcac cggcacgttg agccagaaca cggcgcccca gtggaagaac
4441 tccagcaaca cgccgccgac cagcggaccg atcgcgctgc ccagcgaaaa cgcggcgatc
4501 cacacgccga tggcgaactg gcgctggcgc gggtcgtgga acatgttgcg gaccagcgcc
4561 atggtggacg gcgcgatggt ggcgccggcc aggccgagca aggcgcgcgc cgcgatcaac
4621 agcgcggcgg tatcggccag cgccgcgagc accgaggcga atgcgaagaa cgccgcgccg
4681 atcaacaaca gccggcgccg gccgatgcgg tcgcccagcg tgcccatggt gatcaggaag
4741 ccggcgacga agaagccgta gatgtccagg atccagagaa gctgggcgct ggagggctgc
4801 agttcacggc tgagcaccgg cagcgccagg ttcagcaccg tgaggtccat ggcgtagacc
4861 aggcacggca gggcgaccac ggccaggccg atccattcgc ggcgggtggc cttccgacga

4921 tccgctgcgg tgtcgtggag cgtggcggac atactcaaaa cttccgggac aagaatgctt
4981 taacgacgaa ggcaacgcgc gcatttcgac atgccgtcga tggcaacacc catccgcatt
5041 caggcgcgcg tgacacgcat gcctgttgcc cgtcgcacat cagggggcac ggtcgtagcc
5101 ggccagcagc tgaagtactg acctcatttt ttccttcatt catttcaaac ctctttttaa
5161 tgttttaggt tatcgccagc tcggcaactt gatactctgg tgaagcccaa ctttgtttta
5221 gggcgactgc cctgctgcgt aacatcgttg ctgctccata acatcaaaca tcgacccacg
5281 gcgtaacgcg cttgctgctt ggatgcccga ggcatagact gtacaaaaaa acagtcataa
5341 caagccatga aaaccgccac tgcgccgtta ccaccgctgc gttcggtcaa ggttctggac
5401 cagttgcgtg agcgcatacg ctacttgcat tacagtttac gaaccgaaca ggcttatgtc
5461 cactgggttc gtgccttcat ccgtttccac ggtgtgcgtc acccggcaac cttgggcagc
5521 agcgaagtcg aggcatttct gtcctggctg gcgaacgagc gcaaggtttc ggtctccacg
5581 catcgtcagg cattggcggc cttgctgttc ttctacggca aggtgctgtg cacggatctg
5641 ccctggcttc aggagatcgg aagacctcgg ccgtcgcggc gcttgccggt ggtgctgacc
5701 ccggatgaag tggttcgcat cctcggtttt ctggaaggcg agcatcgttt gttcgcccag
5761 cttctgtatg gaacgggcat gcggatcagt gagggtttgc aactgcgggt caaggatctg
5821 gatttcgatc acggcacgat catcgtgcgg gagggcaagg gctccaagga tcgggccttg
5881 atgttacccg agagcttggc acccagcctg cgcgagcagc tgtcgcgtgc acgggcatgg
5941 tggctgaagg accaggccga gggccgcagc ggcgttgcgc ttcccgacgc ccttgagcgg
6001 aagtatccgc gcgccgggca ttcctggcgg cactgttgca aatagtcggt ggtgataaac
6061 ttatcatccc cttttgctga tggagctgca catgaaccca ttcaaaggcc ggcattttca
6121 gcgtgacatc attctgtggg ccgtacgctg gtactgcaaa tacggcatca gttaccgtga
6181 gctgcaggag atgctggctg aacgcggagt gaatgtcgat cactccacga tttaccgctg
6241 ggttcagcgt tatgcgcctg aaatggaaaa acggctgcgc tggtactggc gtaacccttc
6301 cgatctttgc ccgtggcaca tggatgaaac ctacgtgaag gtcaatggcc gctgggcgta
6361 tctgtaccgg gccgtcgaca gccggggccg cactgtcgat ttttatctct cctcccgtcg
6421 taacagcaaa gctgcatacc ggtttctggg taaaatcctc aacaacgtga agaagtggca
6481 gatcccgcga ttcatcaaca cggataaagc gcccgcctat ggtcgcgcgc ttgctctgct
6541 caaacgcgaa ggccggtgcc cgtctgacgt tgaacaccga cagattaagt accggaacaa
6601 cgtgattgaa tgcgatcatg gcaaactgaa acggataatc ggcgccacgc tgggatttaa
6661 atccatgaag acggcttacg ccaccatcaa aggtattgag gtgatgcgtg cactacgcaa
6721 aggccaggcc tcagcatttt attatggtga tcccctgggc gaaatgcgcc tggtaagcag
6781 agtttttgaa atgtaaggcc tttgaataag acaaaaggct gcctcatcgc taactttgca
6841 acagtgcc
//
